# Review and assessment of policies and practices for spinal cord injury-related health care supplies, services, and mobility equipment reveal wide inequities and inadequacies in Canada

**DOI:** 10.3389/fpubh.2026.1762879

**Published:** 2026-04-13

**Authors:** Peter B. Warkentin, John H. Gregory, Peter Athanasopoulos, Jacquie Ripat, Kristine C. Cowley

**Affiliations:** 1 Department of Physiology and Pathophysiology, Spinal Cord Research Centre, Rady Faculty of Health Sciences, University of Manitoba, Winnipeg, MB, Canada; 2 Spinal Cord Injury Ontario, Toronto, ON, Canada; 3 Department of Occupational Therapy, College of Rehabilitation Sciences, Rady Faculty of Health Sciences, University of Manitoba, Winnipeg, MB, Canada

**Keywords:** attendant services, catheters, health insurance, home care services, public health, universal health care, wheelchairs

## Abstract

**Introduction:**

Canada maintains a universal, publicly funded health care system, although provinces and territories retain flexibility and independence in determining eligibility and funding levels for services and supplies. This flexibility and independence can lead to variability in health care delivery and therefore downstream health outcomes between provinces for the same health condition. Over 80,000 Canadians live with spinal cord injury (SCI), which causes motor paralysis and dysfunctions in multiple body systems. These dysfunctions are managed as multiple chronic health conditions to maintain life after injury. Thus, we identified whether and how a critical subset of health services, supplies, and equipment needed to sustain life after SCI are delivered at the provincial and federal levels for: attendant services for activities of daily living, medical supplies to manage neurogenic bladder and bowel function, and manual and power wheelchairs (WCs).

**Methods:**

A scoping policy review of online documentation identified eligibility criteria, assessment processes, coverage characteristics, quality and quantity limitations, delivery mechanisms, and any quality assurance processes. Key informant (KI) interviews were analyzed descriptively and thematically to verify policy documentation and identify additional documentation when needed, and to indicate gaps between policy and application.

**Results:**

Widespread discrepancies and inadequacies were found in the quality and level of SCI-related health care. Policies indicated means-testing for attendant services in 5/10 provinces; 9/10 for bladder and bowel supplies; and 4/10 for WCs. Public funding is often reduced or withdrawn at very low incomes. Provinces without means-testing loan WCs from restricted equipment pools. KI interviews indicated profound negative implications of stated inadequacies on health and life quality. National programs for Veterans and Indigenous Canadians do not means-test, but eligibility and service restrictions were reported to confer additional hardships on Indigenous Canadians.

**Discussion:**

In contrast to other forms of care (e.g., dialysis for kidney disease), SCI-related health care appears inadequate, highly variable, and focused upon social and financial factors rather than medical need. For those with SCI, age restrictions further limit access to health care. Future research of countries with similar decentralized, multiregional health care systems would aid in developing national standards and best practice delivery mechanisms.

## 1 Introduction

Canada adopted a publicly funded and universally available health care system with the primary objective “to protect, promote, and restore the physical and mental well being of residents of Canada and to facilitate reasonable access to health services without financial or other barriers” (1). Medical services, supplies, and equipment deemed essential—and therefore eligible for funding—are supported through transfer payments to each province and territory in Canada. Each province or territory maintains considerable flexibility and independence in determining items deemed essential and reasonable for funding. Depending upon the disease/condition and type of health care needed, some or all the medical supplies, drugs, and/or equipment required may be funded by provincial health authorities.

For example, essential services such as kidney dialysis and all associated medical supplies are supplied free of charge in Canada without means-testing [wherein each province pays 100% of costs regardless of the person's home province, income, or age (2)]. However, there is often a lack of transparency in the financial decision-making processes and evidence levels required to inform whether a health service, supply, or piece of equipment is publicly funded. Consequently, variability in health care budgets contributes to discrepancies in morbidity and mortality, care, life quality, wait times, and even downstream effects on social costs and public spending. Provision of health care in Canada also appears to differ by disease or injury, despite meeting the criteria of “essential to maintain life”. These discrepancies are often exacerbated in complex chronic conditions such as spinal cord injury (SCI) or disease.

SCI affects over 80,000 Canadians (3) and results in varying degrees of motor paralysis, sensory loss, and a range of autonomic dysfunctions caused by the loss of neural regulation of many bodily tissues and organs. Since there are no curative treatments for SCI, a range of medical services, equipment, and mobility devices are used to manage these essential body functions after SCI. Those with higher level and greater extent of injury lose more function than those with a lower level or smaller extent of spinal cord damage (4). As function and strength differ based on spinal level of SCI, some, but not all individuals living with SCI require assistance in the form of attendant services for activities of daily living (ADL). Typically, those with SCI at spinal C4/C5 or above would require total assistance, and those with injury between C6 and C8 some assistance, depending upon remaining arm and hand function and strength, whereas those with spinal injury at thoracic (*T*) levels or below would be expected to be independent in ADL (4).

The effects of SCI are permanent and often include neurogenic bladder and bowel dysfunction (5, 6), which therefore necessitates other medical management strategies to remove urinary and gastrointestinal wastes to sustain life (4). Wheelchairs (WCs) for mobility are required 100% of the time for those with complete motor paralysis of the legs, which includes all those with severe injury at or above the 2nd lumbar (L) spinal injury level (and likely those with injury between L2 and L5), which represents the vast majority of people with traumatic SCI (4). Thus, attendant services for ADL, neurogenic bladder and bowel supplies, and WCs represent three key domains of health need after SCI—removing or limiting access to these domains would cause significant morbidity and eventual mortality.

Health care management of the chronic conditions caused by SCI is dependent upon the level and extent of each person's injury and requires person-centered, integrated, and individualized services, supplies, and equipment to meet their specific functional needs. Within the context of health equity and rights-based health care, one would anticipate SCI-related health care to be similar within Canada between provinces and at the federal level. Yet those working within the SCI rehabilitation fields as members of national SCI organizations have noted discrepancies between provinces and by funder for identical health care services, supplies, and mobility equipment. During the early stages of the pandemic, these discrepancies became more evident.

As members of a pan-Canadian SCI and COVID response team consisting of SCI-physiatrists, physiotherapists, occupational therapists (OTs), SCI researchers, and SCI community organization members, we met bi-weekly to find solutions to and provide information resources to address emerging and urgent issues related to access to health services, supplies, and equipment within the rapidly changing pandemic situation. We noted that there were substantial discrepancies between provinces and between urban and rural areas regarding vulnerability to workers refusing to provide in-home attendant services due to pandemic concerns, inability to obtain needed neurogenic bowel management supplies (e.g., gloves), and access to rehabilitation professionals for attaining needed WC equipment. As a result, we identified a need to systematically examine whether and how public funding for essential medical supplies and services to manage key chronic conditions caused by SCI varies between provinces within Canada.

A systematic by-province review of policy documentation relating to these key domains of health care has not been performed in Canada. Thus, we focused on key predictable and lifelong functional losses and investigated policies relating to: (1) attendant services for ADL; (2) medical supplies for managing neurogenic bladder and bowel function; and (3) manual and power WCs and transfer devices. Given the paucity of information regarding public provision of SCI-related medically necessary health care items within the context of a Canadian universal health care system, our objectives were to perform a systematic environmental policy documentation scan and conduct a series of semi-structured interviews of key informants (KIs) in each Canadian province. The systematic online review enabled comparison of relevant policies, identified differences in public funding support, and determined financial implications for those living with SCI in Canada. The KI interviews were descriptively and thematically analyzed to identify gaps between policy and practice, and to provide information on personal life quality implications relating to levels and extent of public health care funding within each domain for those with SCI.

## 2 Methods

Our general approach was to combine policy review with KI interview data relating to funding levels and processes regarding public provision of essential health services and supplies in our identified domains: attendant services for ADL; neurogenic bladder and bowel management supplies; and WCs and transfer devices. We met with the National Executive Committee of Spinal Cord Injury Canada between May and September of 2021 in strategic planning meetings to receive input regarding our selection of domains of essential need and to refine and validate our rationale for selecting items within each domain. Spinal Cord Injury Canada (formerly called the Canadian Paraplegic Association, with branches incorporated in each province) is a non-profit, non-governmental organization committed to assisting people living with SCI to achieve independence, self-reliance, and full community participation through SCI related information, rehabilitation, and advocacy services (7).

### 2.1 Policy review

We examined each province's documentation regarding eligibility criteria, assessment processes, types and quantities/levels of coverage, delivery mechanisms, and any processes for addressing concerns within each domain. In parallel, data from KI interviews were used to assess and compare relevant public policies in each jurisdiction. This approach allowed us to gather public policies and then identify gaps between policy and experience within our three domains of essential health care. Study procedures used for KI interviews were reviewed and approved by the University of Manitoba Health Research Ethics Board (Ethics #: HS25227/H2021:377). Interview questions can be found in Supplementary Appendix 1.

Health policies were reviewed online between September 2021 and January 2022. The policy review process began prior to the interviews to provide relevant background information and identify local terminology used in health care delivery for each domain to help inform KI interviews. Policy review researchers worked collaboratively to provide information and digital documentation via shared Microsoft Teams documents which were then compiled by province and made available for verification and reference throughout the study by research team members.

Publicly available policy documentation was searched online with relevant key words (e.g., home care manual; medical supply regulation; assistive technology; wheelchair program). Our primary data sources were relevant government health department websites, although for neurogenic bladder and bowel supplies and WCs, online information was much more fragmented, often requiring an iterative process of repeat communication with KIs and use of multiple sources in each province to gather policy information. As can be seen from Table 1, our use of an iterative process enabled identification of primary data sources consisting almost entirely of provincial health websites for each domain. Additional iterative processes were used to validate online information and reduce the likelihood that our findings arose from missing, outdated, or ambiguous policy information, and are described further in Section 2.2 and 2.3. Finally, in the cases of neurogenic bladder and bowel supply programs in MB and BC and of WC programs in BC, communication with additional key SCI in-patient physiatry or high-level SCI organization input was sought to ensure our assessment was accurate.

**Table 1:** Primary source(s) of policy documentation for each domain of SCI-related functional need, by province.

Province	Attendant care	Neurogenic bladder and bowel supplies	Essential mobility equipment
British Columbia (BC)	British Columbia Ministry of Health (72)	Government of British Columbia (73); British Columbia Employment & Assistance (18)	British Columbia Employment & Assistance (29)
Alberta (AB)	Alberta Health Services (74, 75)	Alberta Health (117)	Alberta Health (32, 117)
Saskatchewan (SK)	Saskatchewan Ministry of Health (76)	Saskatchewan Ministry of Health (22)	Saskatchewan Ministry of Health (34)
Manitoba (MB)	Winnipeg Regional Health Authority (77)	Manitoba Department of Families (19)	Manitoba Possible (33)
Ontario (ON)	Centre for Independent Living in Toronto (13)	Government of Ontario (78)	Government of Ontario (30)
Québec (QC)	Gouvernement du Québec (79)	Gouvernement du Québec (134)	Gouvernement du Québec (80)
New Brunswick (NB)	Social Supports New Brunswick (81)	New Brunswick Department of Social Development (27)	New Brunswick Department of Social Development (35)
Nova Scotia (NS)	Nova Scotia Department of Health and Wellness (82)	Nova Scotia Department of Community Services (83)	Nova Scotia Department of Community Services (83)
Prince Edward Island (PEI)	Health PEI (84)	AccessAbility Supports (110)	AccessAbility Supports (31)
Newfoundland & Labrador (NL)	Newfoundland and Labrador Department of Health and Community Services (111)	Newfoundland and Labrador Department of Health and Community Services (25)	Newfoundland and Labrador Department of Health and Community Services (25)

Online sources of data are indicated, with the version year indicated when initially accessed. Note that these sites may have been updated with new dates since initial review.

Health policies were reviewed online between September 2021 and January 2022. All online policies were re-accessed in January to March 2024 to update links to webpages where possible (only 1 of 153 remained broken). For attendant services for ADL, provincial governmental sources were used. Sources were validated and updated January 12, 2026, and online links can be found in Supplementary Table A2. Tables documenting sources for neurogenic bladder and bowel supplies and mobility equipment are found within the Results. Reiterative efforts were made to identify all relevant publicly available information within each province, and tables indicate instances where policy documentation beyond a brief program overview could not be found. This occurred only within the mobility equipment domain in QC and NS.

### 2.2 Key informant interviews

KIs were interviewed using a semi-structured questionnaire format (Supplementary Appendix 1) by one experienced interviewer. Video interviews were recorded and stored securely online. The questionnaire was designed to collect standardized, descriptive information specific to each province in Canada regarding provision of attendant services, medical supplies, and assistive mobility devices via public (provincial and federal) and insurance-based systems. Transcripts were first analyzed descriptively with each KI's response summarized by question in an Excel table, with quotations as appropriate. Summarized data was then analyzed thematically. The semi-structured format facilitated collection of data outside of our specific questions which helped to identify themes highlighted in the Section 3, Results.

We recruited up to three KIs in each province, depending upon their degree of expertise in assisting within each investigated domain. The semi-structured questionnaire included questions to evaluate and assess each KI's level of knowledge regarding each domain (Supplementary Appendix 1). Table 2 summarizes each KI's professional expertise, frequency of activities relating to policy and/or delivery of SCI-related supplies, services, and equipment, and areas in which they indicated a lack of knowledge. The KI interviewer made an initial assessment of knowledge level for each KI and brought it to one of the research team for discussion to determine if further KIs were required for that jurisdiction. KI recruitment continued until all questions were adequately answered and/or until data saturation for that province was reached. One to three KIs were interviewed for each province to obtain a critical review of the existing policies and practices in their jurisdiction (referred to with a subscript identifier indicating province). To increase completeness of our policy documentation, we requested that KIs forward all policy documentation they used to the research team. If it contained new sources or information, we added it to our spreadsheets and compared it to our existing documentation and KI responses. If needed, we followed up with additional email queries as noted in Section 2.3.

**Table 2:** Summary of key informant expertise, professional experience, and knowledge gaps noted during semi-structured interviews.

KI	Relevant employment role(s)	Lived SCI? (Y/N)	Frequency of involvement in obtaining services, supplies, or equipment	Domain/topic KI declined to comment on due to lack of knowledge
1	Former executive director of a provincial SCI organization. Expertise in program/systems planning level, policy analysis; advocating for policy revision.	Y	Daily	NA
2	Multiple roles with a provincial SCI organization, including peer support coordination, services and program management, policy and legislation development, community engagement, and personal interaction.	Y	Daily	NA
3	Executive director and director of services with a provincial SCI organization. Duties include consultation for frontline staff, helping individuals achieve transportation goals, housing, equipment, supplies, medications, and recreation.	N	~3 days/week	NA
4	Two roles with a provincial SCI organization (hospital counselor and assistant director of rehabilitation services).	N	Weekly	NA
5	Client service coordinator with a provincial SCI organization. Duties include information services, system navigation, accessing equipment, supplies, funding, and services.	Y	Daily	Attendant services restrictions; supply quantity limits; independent living philosophy
6	Client services and peer support coordinator with a provincial SCI organization. Registered social worker. Duties include aiding clients accessing equipment, supplies, and care.	Y	Weekly	Independent living philosophy
7	Manager of an SCI resource centre with a provincial SCI organization.	Y	Weekly	Delegated care and cost-sharing for attendant services; catheter supply restriction; mobility assistive device areas. Referred to KI 10
8	Director of client services with a provincial SCI organization. Oversight of all client services.	N	Daily	WC model restrictions
9	Social worker with a provincial SCI organization. Duties include client inquiries and funding options.	N	Weekly	NA
10	Peer support specialist with a provincial SCI organization. Liaison to community health, including accessing funding and supplies.	Y	Daily	NA
11	Executive director and former counselor with a provincial SCI organization.	Y	Weekly	NA
12	Multiple: board member with SCI organizations at national and provincial levels.	Y	Weekly	Delegated care; assessment; appeals; self-management options for attendant services
13	Peer counselor with a provincial SCI organization.	Y	Weekly	Independent Living Philosophy
14	Integration advisor with a provincial SCI organization.	Y	Weekly	Service delivery models; assessment; hospital discharge for attendant service. Referred to KI 13
15	Indigenous services counselor with a provincial SCI organization. Duties include assessment, client connection for medical, home care, rehabilitation, and housing needs, self-advocacy education, and attending medical appointments with clients.	N	~10 h/week	Inpatient assessment for attendant services
16	Occupational therapist in acute care and non-profit community-based rehab service delivery setting. Duties include equipment and assessment consultations. Expertise in mobility and other assistive technologies.	N	Weekly	Modified interview was focused on equipment

KIs were selected based on their expertise with these programs and had experience in the field of SCI rehabilitation. KI study participants were either executive directors, board members, rehabilitation counselors, social workers, OTs, or peer support and service coordinators employed by provincial non-governmental, non-profit SCI and/or disability organizations. To facilitate data collection within Québec, a bilingual (French/English) interviewer with expertise in SCI rehabilitation assisted during KI interviews and in fact-checking our translated reports.

A total of 19 interviews with 16 KIs (*n* = 10/16 also had lived SCI experience) were performed between October 2021 and May 2022. Three KIs were interviewed twice to clarify data collected during their initial interview. Interviews were transcribed and summarized by the study interviewer, and data was collated with the policy information and fact-checked by research team members. KIs received a small honorarium for their participation in the interview process.

### 2.3 Data handling and analysis

All KI participants completed the study and all those invited agreed to participate. Personal information was kept strictly confidential, and all recordings, transcripts, and documents were stored electronically using Microsoft Teams before and after being transcribed. Personal identifiers were removed from the transcriptions, and transcripts were coded for reference and identification. Only research staff had access to original transcripts.

Policy documentation for each province was compiled in tables with column headings corresponding to the semi-structured KI interview questions to facilitate direct comparison and analyses for each domain. An iterative process was used to triangulate data between policy documents and KI information in each province. Researchers also confirmed and verified information in multiple email communications with KIs as needed throughout the data summary and analysis process to address any conflicting information identified. As a final step, each KI interview summary was sent back to each participant to verify their information had been captured and summarized accurately.

There was considerable overlap between policy documentation and descriptions from KI interviews. For the most part, interview findings and KIs' opinions were supported by data gathered in the environmental scan. In areas where KI expertise was lacking, relevant data gathered from public policy were used to fill in knowledge gaps. In cases where public policy contradicted KI interview data, all contradictions were included in our iterative provincial summary process and sent back to KI for review and clarification. In each case, KIs indicated that to the best of their knowledge, our interpretations of provincial policy were accurate.

The data tables generated were then used to identify significant differences and themes between provinces in policies and between KIs' descriptions of practices within each province. After completing the descriptive analysis, themes were identified and documented in a spreadsheet. Verbatim transcripts were analyzed for recurring themes, and when a minimum of three provinces reported a similar theme, these were summarized and documented (Table 10). Themes were discussed between team members and at monthly team meetings.

Interview and documented policy data were also used to analyze financial implications of means-testing (i.e., the assessment of the person's or family's ability to pay for a particular medical service, supply, or piece of equipment) within each domain. An initial report of the findings was written in English and French and sent directly to the funder Praxis, each KI, and each provincial chapter of the Spinal Cord Injury Canada federation of non-profit, non-governmental organizations for their own use (8).

### 2.4 Person-centered financial implication analyses

After our initial analysis of policies relating to means-testing and financial assessments for determining co-pay levels, it became apparent that a wide range of financial calculation policies existed. This was most evident within the domain of attendant services for ADL, in which 5/10 provinces used means-testing to determine cost-sharing requirements between the health system and the individual receiving services. As such, we performed a simple *post-hoc*, person-centered financial analysis of the means-testing and funding eligibility criteria to compare financial implications between provinces.

Means-testing was reviewed by documenting online policies outlining financial assessment formulae and then performing mathematical calculations to determine financial eligibility, cut-off levels, and inflection points for co-pay fees using the case scenario of a single adult (aged 18–65) with SCI dwelling alone in the community as our ‘model' for each province. Online formulae were used to determine income levels at which public funding for health care services, supplies, or equipment would be withdrawn for each domain. Modeling other scenarios and performing a related sensitivity analysis was outside the scope of this paper.

We also identified whether delivery was dependent upon hours of need for attendant services for ADL. For neurogenic bladder and bowel supplies, we identified if limits existed in the quantity or quality of supplies delivered. For WCs and transfer devices, we identified if limits in available models or necessary sub-components existed in medical mobility and transfer device equipment delivery.

## 3 Results

Overall, we observed widespread discrepancies and inequities in public health care delivery within each domain of essential SCI-related medical need. All provinces required financial assessment to determine eligibility for public health care delivery in at least one and most often two or three domains (Table 3). Only federal health care programs (Indigenous Services Canada, ISC; Veterans Affairs Canada, VAC; and the Veterans Independence Program, VIP) funded items in all domains without means-testing, thereby meeting the definition of universal access to essential health care as described in the Canada Health Act (1).

**Table 3:** Summary of means-testing for each domain of SCI-related functional need.

Province/Program	Means-tested attendant services for ADL	Means-tested medical supplies to manage neurogenic bowel and urinary dysfunction	Means-tested manual and power wheelchairs
BC	Yes	Yes	Yes
AB	No	Yes	No
SK	Yes	No	No
MB	No	Yes	No
ON	No	Yes	Yes
QC	No	Yes	No
NB	Yes	Yes	No
NS	Yes	Yes	No
PEI	No	Yes	Yes
NL	Yes	Yes	Yes
FNIHCC/NIHB	No	No	No
VAC	No	No	No

Public funders that use financial assessments to determine eligibility to receive reimbursement for, or provision of, attendant services for ADL, neurogenic bowel and bladder management supplies, or wheelchair equipment are indicated with Yes.

ADL, activities of daily living; FNIHCC, First Nations and Inuit home and community care; NIHB, non-insured health benefits for First Nations and Inuit; VAC, Veterans Affairs Canada.

Our systematic review also investigated public, worker's compensation, and vehicle insurance-based funding for each domain of SCI-related essential need in each province, but their description is outside the scope of this manuscript. In almost all cases, the public funder was the ‘funder of last resort' such that if a person's injury arose through a motor vehicle or workplace accident, their SCI-related needs were typically met through those compensation programs, and the person became ineligible to receive services, supplies, or equipment through the public provider. Generally, insurance-based schemes funded additional items such as home renovations, and better quality supplies, services, and equipment with a wider range of options and more timely access to WC equipment and neurogenic bladder and bowel supplies than what was available through provincial public funders.

Access to health care was limited in that means-testing reduced or eliminated eligibility to receive any publicly funded health care in 5/10 provinces for attendant services; in 9/10 provinces for neurogenic bladder and bowel supplies; and in 4/10 provinces for WCs (Table 3). A loans-based equipment pool was used to provide WCs in the six provinces that did not means-test to limit eligibility for public funding of WCs for a person with SCI. Overall, within each province, whether public funds contributed to these essential SCI-related medical items appeared to be determined largely by social and economic factors rather than physical need or functional criteria.

### 3.1 Attendant services

#### 3.1.1 Provincial discrepancies in public funding contributions and organization of attendant services provision for activities of daily living

In Canada, levels of public funding, inclusion and exclusion criteria, and processes to obtain attendant services vary widely. Policy documentation was publicly available for each province (Supplementary Table 2), indicating some level of public funding for attendant services is provided, and that restrictions and limitations in available services, means-testing, and co-pay requirements vary widely between provinces (Table 4). Generally, delegation of physically invasive tasks (e.g., neurogenic bowel management) to reduce service delivery costs is allowed as outlined by provincial health legislation and/or nursing standards of practice. Policy documentation also indicated federally managed attendant service programs for Indigenous Canadians and Veterans with SCI are not means-tested. Veterans with SCI apply to the VIP and are assessed to determine their health-related needs and identify appropriate services that support self-sufficiency within the home (9). Currently, significant delays are reported within the VIP, with wait times up to 18–24 months for assessment (KI_PEI_).

**Table 4:** Summary of financial implications for the 5/10 provinces using means-testing in their calculations of attendant services fees reveals widespread discrepancies in coverage levels.

Province	Attendant services financial implication modeling for a person living with SCI
BC	Fees are based on **income**, not hours of need. **Calculation:** “Remaining annual income” is calculated using a formula involving net family income, family size, income tax, and child-care benefits, and then multiplied by 0.00138889 (if >$41,511) to determine a daily rate. **Annual maximum:** if remaining income >$50,000, maximum is **$3,600** in fees.
SK	Fees are based on **hours of need and income**, with a sliding scale of fees. **Calculation:** $9.60/h for first 10 h, then a detailed formula is used to calculate fees based on net family income, a basic income exemption, and a sliding scale of hourly fees for additional hours of care received. The basic income allowance is $1,907 per month ($22,884 per year). For earnings above this limit, additional home care rates apply. For example, a single person earning >$22,884 annually requiring 15 h per week is assessed fees of 100% of additional income (i.e. beyond their allowable monthly allowance of $1,907) for income ≤ $30,072 annually. The same person requiring 10 h per week of care is assessed fees of 100% of additional earned income ≤ $25,884 annually. **Annual maximum:** for earnings >$30,072, a single individual requiring 15 h per week of care would pay the yearly maximum of **$6,912** in attendant service fees. This same individual would pay $4,867.20 for 10 h per week of care in fees.
NB	Eligibility and fees are based on **income**, not hours of need, and is subsidy-based. **Calculation:** no fees on earnings up to the social assistance level ( ≤ $10,632/yr). 5% on annual income from $10,632 to $21,624 (~current Old Age Security and Guaranteed Income Supplement Maximums); 30% on income from $21,624 to $25,000; 100% on annual income >$25,000. Thus, a single person earning $25,000/year is assessed at $1,562.40 annually. If earning $50,000, calculated fees are $26,562.40 annually. **Annual maximum:** no stated maximum (any income >$25,000/yr is paid as attendant care fees). Individuals pay annual fees regardless of hours of attendant care required.
NS	Fees are based on **hours of need and income**. **Calculation:** no fees on earnings up to $30,797/yr. Hourly rates are determined using income-based sliding scale. Thus, a single person receiving services with annual income from $30,798 to $45,797 fees is assessed at $12.45/h up to a monthly maximum of $124.50 ($1,494/yr). For the same person earning from $45,798 to $55,797 service fees are assessed the same hourly rate, up to a monthly maximum of $249 ($2,988/yr); for income from $55,797 to $65,797 fees are capped at $373.50 ($4,482/yr); for income from $65,798 to $75,797 fees are capped at $498 ($5,976/yr) and for income >$75,797 monthly fees peak at $622.50 ($7,470/yr). **Annual maximum:** for single individuals with annual income >$75,797/yr, fees = **$7,470**.
NL	Fees are based on **income**, not hours of need. **Calculation:** no fees on earnings ≤$13,000/yr; 24% on income from $13,001 to $18,000; 34% on income from $18,001 to $23,000; 42.8% on income from $23,001 to $28,000; 18% on income from $28,001 to $150,000. **Annual maximum: $27,000/yr** (single individual earning $150,000/yr). Single individuals earning >$150,000 are not eligible for a home care subsidy.

For ease of comparisons, main assumptions, calculations, and maximums that would be paid for attendant services are based on calculations for a single person living alone with SCI. Additional inclusion and exclusion criteria, limitations, and funding allowances are used to calculate eligibility and funding levels for persons with SCI in other living situations (e.g. in a family, with multiple incomes). ^*^Income, as defined by each province's documentation, often uses detailed formulae that differ between provinces (sources listed in Supplementary Appendix A2). ADL, activities of daily living; SCI, spinal cord injury; FNIHCC, First Nations and Inuit home and community care; VAC, Veterans Affairs Canada; VIP, Veterans Independence Program.

Sources: Government of British Columbia (10); Alberta Health Services (74); Saskatchewan Ministry of Health (76); Manitoba Health (14); Government of Ontario (85); Gouvernement du Québec (79); New Brunswick Department of Social Development (24); New Brunswick Department of Social Development (86); Government of Canada (87, 88); Nova Scotia Department of Health and Wellness (82, 89); Health PEI (84); Newfoundland and Labrador Department of Health and Community Services (25); Indigenous Services Canada (12); Veterans Affairs Canada (9, 90–92).

#### 3.1.2 Means-testing, eligibility cut-off levels, and co-pay attendant care fees

Table 4 indicates funding models and rates of co-pay fees vary widely in each of the five provinces that means-test to determine eligibility for public support for attendant services for ADL (BC, SK, NB, NS, and NL). Although we did not perform a sensitivity analysis, review of documentation and calculators indicated that generally, adding more family members (without increasing family income) would decrease co-pay fees, while adding family members that increase family income would increase co-pay fees.

Publicly available online calculators and documentation that we reviewed and used in our modeling indicates that formulae for calculating co-pay fees are variable, complex, involve multiple levels of information and assessment prior to receiving services, and may involve frequent financial reassessments to retain services and determine ongoing co-pay fees. For example, maximum attendant care fees paid by a single individual with SCI living alone range from $3,600/year in BC to $27,000/year in NL, to 100% of earnings paid on income >$25,000/year in NB (Table 4). Some provinces based their co-pay fees on income alone (BC, NB, and NL) whereas others use income and hours of attendant services needed. Thus, those with more service needs pay higher fees (SK and NS). BC is the only province that uses an inverted fee model with a yearly maximum deductible, such that a single person living alone can earn up to $41,511/year before they are required to pay attendant care fees (10). Co-pay fees then increase linearly with income until earning $50,000/year, at which point a maximum yearly fee of $3,600 is assessed (7.2% of income). Thus, comprehensive calculations from policy documentation demonstrate the wide range of co-pay fees required and the relatively high co-pay fees required in NS, NB, NL, and SK (Table 4).

Public policy documentation indicated that Canadians with SCI eligible for services through VAC must first access provincial and community programs before receiving services through the VIP (9). Coverage of home care services provided through the VIP has an annual maximum of $11,842.40, although this amount may be exceeded in exceptional circumstances (11). Although access to attendant services for Indigenous Canadians and Veterans with SCI is not restricted based on financial eligibility, KI indicated difficulties arise based on location of needed services and staffing inadequacies (KI_MB_).

KIs in all provinces report significant barriers and complications when attempting to receive services, change service levels, or move within or between provinces. Although federal programs do not means-test to assess co-pay fees for attendant services for ADL, other restrictions apply. For example, eligibility for the ISC home care program requires First Nations or Inuit membership or citizenship, to be living in a First Nations or Inuit community, and to have undergone a formal assessment of needs (12). Indigenous clients living outside of such communities must access home care services through provincial or private programs.

#### 3.1.3 Provincial discrepancies in available services

Policy documentation indicated that provinces such as ON allow attendant services at places of employment (13) whereas other provinces (e.g., MB) only provide services at home (14), which KI_MB_ reports can interfere with a person's ability to find or maintain employment. Other provinces implement very low income cut-offs before co-pay fees are required, typically at levels high enough that employment seems untenable, particularly for those entering the workforce at entry-level positions (Table 4).

A common theme that arose from KI interviews (BC, ON, NB, and NS, Table 10) was the lack of properly trained personal support workers and a lack of accountability. In ON, attendant services are provided by regional health authorities, often with a mix of public and private service delivery. KIs indicated that clients frequently report missed visits by attendant care workers, particularly with private service providers, and that this trend worsened during the pandemic.

KIs also indicated a lack of proper accountability and documentation regarding numbers of missed service visits, and/or that companies contracted to provide personal attendant services demonstrating large numbers of missed visits were not held accountable for inadequate service delivery during renegotiation of service contracts (KI_ON_). Lack of quality control also appears in the other domains of SCI-related health care (see further below regarding catheters and WCs).

‘‘*The safety issues in New Brunswick arise due to the home support worker shortage. We have a lot of people that have workers that aren't showing up.” KI*_NB_

#### 3.1.4 Indigenous persons faced unique additional challenges such as a lack of sanitary and accessible housing and access to clean water

For Indigenous Canadians, eligibility for coverage through ISC is dependent on First Nations or Inuit membership (12). Although federally managed attendant service programs for Indigenous Canadians with SCI are not means-tested, KIs report that eligibility requirements for coverage pose a significant concern, as Indigenous people do not have control over their own membership and some community members may have lost or never had Indigenous status (KI_MB_), and are therefore ineligible to receive services through this program. Our KI also reported that it remained unclear as to which public funder was required to cover specific items and services, which led to further delays in health care provision. For Indigenous persons with SCI, access to properly trained attendant service workers was also reported as an issue because of a lack of available trained service providers. This issue was exacerbated by inadequate housing and an ongoing absence of clean running water in First Nations and Inuit communities [(15), KI_MB_]. Some Indigenous clients reported a commode in their bedroom as a bathroom, and for many, sponge bathing as the norm (KI_MB_). Indigenous community members report not being comfortable going to an urban center for training (KI_MB_). As a result, there is a lack of trained people available for hire within Indigenous communities. Often, Indigenous persons with significant SCI-related care needs report their only option if they wish to remain in their home community is to live in a long-term care facility intended for older adults (where available), regardless of their age (KI_MB_). Combined, these challenges pressure Indigenous persons with SCI to move to urban centers to receive essential health care, where they may be physically isolated and lose the emotional and social support(s) of their home community (KI_MB_).

#### 3.1.5 Alternate flexible models of attendant service delivery in Canada

Review of public policy documentation and KI reports indicated that many provinces have alternate delivery models for attendant services, consistent with a previous review of directly-funded attendant service care in Canada (16). KIs reported that the historical impetus for the development of these alternate care models arose as a response to repeated requests by persons living with disabilities and organizations that represented them (e.g. SCI organizations and Independent Living Resource Centers) which understood that enabling employment and community contributions required persons with disabilities to receive attendant services with flexibility in scheduling and options (KI_MB_, KI_ON_). As such, self-managed attendant service delivery exists in some form in each province (Table 5).

**Table 5:** Summary of attendant services for ADL by provincial or federal jurisdiction: alternative delivery models.

Province/ Program	Alternative attendant service arrangements
BC	**Self-managed attendant care (CSIL):** no maximum coverage is stated. Funding calculated by multiplying CSIL hourly rate by assessed hours of need. KIs indicated those with very high needs are often encouraged to seek out long term care facilities.
AB	**Self-managed attendant care:** no maximum coverage is stated. KIs indicate those in rural settings may be limited to self-managed care. Cannot hire family members. **Family-managed attendant care**. as above for self-managed. **Indirect funding:** community-based group supportive living (Designated Supportive Living).
SK	**Self-managed attendant care:** (Individualized Funding): monthly maximum coverage of $7,474. Coverage is based on assessed hours of care needed. Cannot hire family. **Indirect funding:** collective funding arrangement exists for groups of people living together if eligible for self-managed care.
MB	**Self- and family-managed attendant care:** accessed through the Center for Independent Living in Winnipeg. No maximum coverage is stated. Family members are not allowed to be hired except in unique circumstances. **Indirect funding:** community-based supportive housing models, e.g. Fokus Group.
ON	**Family-managed home care:** through Home and Community Care Support Services. **Self-managed attendant care:** accessed through Direct Funding via the Center for Independent Living in Toronto. Hours are negotiated individually (maximum 7 h/day, 212/month). **Attendant outreach services:** expanded services outside of the home. **Indirect funding:** community-based supportive living. **Other:** emergency attendant service support available in Toronto/York.
QC	**Self-managed attendant care** (L'allocation directe/chèque emploi-service): no maximum coverage stated. Clients are allowed to hire family members. **Indirect funding:** community-based supportive living exists as special cases.
NB	**Self-managed attendant care:** hiring of family members not living in the same household is allowed. No maximum coverage is stated. KIs indicated those with very high needs are often encouraged to seek out long term care facilities. **Indirect funding:** community-based supportive living options exist (e.g. shared housing).
NS	**Self-managed attendant care:** KI stated that many clients with high care needs apply for self-managed care as scheduling flexibility is increased. Maximum 100 h of home support per 28-day period.
PEI	**Self-managed attendant care:** AccessAbility Supports program provides a range of supports. Monthly maximum allowance can be directed to the individual's specific needs. It is unclear if self-managed attendant services could be supported by this program. **Indirect funding:** community-based supportive living (e.g. Kay Reynolds Center).
NL	**Self-managed attendant care:** can only hire family members in exceptional circumstances. Maximum coverage not stated. **Indirect funding:** community-based supportive living (Shared Living Arrangements).
ISC	**Assisted living program:** non-medical social support services can be provided in-home.
VAC	**Attendance allowance:** direct funding for personal caregiver support in ADL.

ADL, activities of daily living; CSIL, choices in support for independent living; ISC, indigenous services Canada; VAC, veterans affairs Canada; KI, key informant.

Sources: British Columbia Home and Community Care (93); British Columbia Ministry of Health (72, 94); KI_BC_; Alberta Health Services (75); KI_AB_; Alberta Health (95); Saskatchewan Ministry of Health (76, 96); KI_SK_; Independent Living Resource Centre (97); Winnipeg Regional Health Authority (77); Ten Ten Sinclair Housing (98, 99); Home and Community Care Support Services South West (100); Centre for Independent Living in Toronto (13, 101); Gouvernement du Québec (79, 102); KI_QC_; KI_NB_; Social Supports New Brunswick (81); Nova Scotia Department of Health and Wellness ((82, 103–105)); KI_NS_; AccessAbility Supports (23, 31, 106–110); KI_PEI_; Newfoundland and Labrador Department of Health and Community Services (111–113); Indigenous Services Canada (114); Veterans Affairs Canada (115).

Another model that evolved which enables those with relatively high attendant service needs to live independently in the community is a shared service provider model, in which several persons live in close proximity (e.g., within the same apartment building) for increased service access and efficiency. Although these different forms of attendant services increase flexibility and enable employment, education, and community contributions, they are subject to the same financial considerations as mainstream attendant service delivery (Table 5).

### 3.2 Neurogenic bowel and bladder supplies

#### 3.2.1 Public health care delivery for life-sustaining neurogenic bladder and bowel supplies provided in 1/10 provinces; none comply with best practice recommendations of the Canadian Urological Association

Policy documentation available for each province indicated that currently, SK is the only province in Canada that does not restrict access to neurogenic bladder and bowel management supplies based on income (Table 3) (17). Supplied catheters are limited by quantity and quality in each province and require physician prescription. Means-testing is applied inconsistently between provinces regarding support for catheters (Table 6). Policy documentation indicates 100% coverage for neurogenic bladder and bowel supplies is only available to those receiving social assistance in BC, MB, and ON (18–20). There is no funding for these supplies if/when the person does not receive income assistance [(18–20), KI_ON_]. In AB, clients pay 25% of supply costs up to an annual maximum of $500 (21), while SK is the only province with 100% coverage of medical supplies for SCI-related bowel and bladder management supplies regardless of income (22).

**Table 6:** Summary of neurogenic bowel and bladder management supplies: means-testing, financial implications and quantity/quality limitations, by regional jurisdiction.

Province/ Program	Summary of neurogenic bowel and bladder management supplies: means-testing, financial implications, and quantity/quality limitations, by regional jurisdiction
BC	**Eligibility:** medical prescription, financial assessment, and limited to **those receiving EAPD** (with annual income for single individual ≤$16,200). **Single-use catheterization:** not supported; quantities limited (unless medically justified).
AB	**Eligibility:** medical prescription and financial assessment. 100% coverage if earning < $20,971/yr; 75% coverage if earning ≥$20,971/yr. Individuals cover remaining 25% of costs up to maximum of $500/yr. **Single-use:** not supported; limited to 35/month without medical justification.
SK	**Eligibility:** medical prescription, confirmation of paralysis, and ineligibility for funding through other government agencies. **Single-use:** not supported; quantities limited (4 catheters per day; limit may be exceeded with medical justification).
MB	**Eligibility:** medical prescription, financial assessment, and the individual **must be receiving EIA or home care for ADL** (although this does not guarantee provision). **Single-use:** not supported; quantities limited.
ON	**Eligibility:** medical prescription and financial assessment: supplies provided only to **those receiving income support through ODSP**. Income limits not indicated. **Single-use:** not supported; quality and quantities limited (~1/day without medical justification).
QC	**Eligibility:** medical prescription and financial assessment. Supplies **provided for those with very low income and significant need**. Income limits not defined. **Single-use:** not supported; limited quality and quantities (medical justification to exceed).
NB	**Eligibility:** medical prescription and financial eligibility, which is reviewed on an individual basis and requires a “budget deficit”. **Not eligible if earning** **>~$25,000/yr**. **Single-use:** not supported; quantities are limited (4 intermittent catheters per day, 4 indwelling per month, may be exceeded with medical justification).
NS	**Eligibility:** medical prescription and financial eligibility, reviewed on an individual basis. No specific income limits. Assessment includes income, assets, and associated costs. **Applicants must apply all income toward costs of supports** provided through the Disability Support Program (numerous exemptions may apply, including a $300/month exemption, GST tax credit, etc.). **Single-use:** not supported; limited quality and quantities.
PEI	**Eligibility:** through AccessAbility Supports, a single person would pay $0 for medical supplies up to an annual income of $21,707. Fees then assessed at 10% of relevant costs for income ≤$51,707, and fee percentages would then increase for income ≤$119,707. Ineligible for medical supplies with income >$119,708. **Single-use:** supported if justified and copays are covered.
NL	**Eligibility:** based on calculation of “Needs Test” which involves consideration of income, RRSP/RRIF contributions, and allowable expenses (~20 are specified). Calculations are complex, require professional involvement and adherence to a flowchart with multiple decision points. **Single-use:** not supported. Typically limited to one catheter/week.
FNIHCC/NIHB	NIHB is a “supplier of last resort” such that supplies are provided only if the person is not eligible through private/public insurance or social programs. Medical supply provision through FNIHCC is collaborative with other social programs including NIHB. NIHB eligibility requires that a client is a First Nations person registered under the *Indian Act*, an Inuk recognized by an Inuit land claim organization, or a child < 2 years old whose parent is an NIHB client. **Single-use catheterization:** not supported, quality and quantities limited (4/day, restriction may be waived if living in areas without access to clean running water).
VAC	Medical supplies available to those who qualify for VIP or Disability Benefits through VAC. Requires prescription and preauthorization. Cost and frequency restrictions outlined in relevant benefit grids. If a prescription exceeds these limits, prescription/communication with a doctor or relevant health care professional is required. Items not appearing on benefit grids but are prescribed and justified may also be approved. **Single use:** catheter supplies ≤$350 per month are eligible, separate allowances for urostomy supplies, disposable gloves, and “other” related supplies.

For ease of comparisons, main assumptions, calculations, and maximums that would be paid for attendant services are based on calculations for a single person living alone with SCI.

ADL, activities of daily living; SCI, spinal cord injury; EAPD, employment and assistance for persons with disabilities; EIA, employment and income assistance; ODSP, Ontario disability support program; RRSP, registered retirement savings plan; RRIF, registered retirement income fund; FNIHCC, First Nations and Inuit home and community care; NIHB, non-insured health benefits for First Nations and Inuit; VAC, veterans affairs Canada; VIP, veterans independence program; KI, key informant.

**Sources:** British Columbia Employment & Assistance (18); British Columbia Ministry of Health (116); KI_BC_; Government of British Columbia (73); Alberta Health (117, 118); Saskatchewan Ministry of Health (22); Manitoba Department of Families (19); KI_MB_; Government of Ontario (78, 119); KI_ON_; Gouvernement du Québec (120); KI_QC_; New Brunswick Department of Social Development (27); Government of New Brunswick (121); KI_NB_; Nova Scotia Department of Community Services (83); AccessAbility Supports (23); Newfoundland and Labrador Department of Health and Community Services (25, 122); KI_NL_; Indigenous Services Canada (15, 123, 124); Veterans Affairs Canada (125–127).

Policy documentation summaries in Tables 6 and 7 indicate coverage for supplies in the remaining provinces (QC, NB, NS, PEI, and NL) only for those with very low income, with specific income limits clearly stated for NB, PEI, and NL (23–25). In addition to limiting quantities, many provinces (BC, AB, MB, ON, QC, NB, and NS) and the ISC program stipulate that the quality of supplies be limited, typically to the least expensive option available (Table 7). In summary, with the exception of AB and SK, funding for neurogenic bladder and bowel supplies is restricted to those Canadians with very limited income, in many cases below Canadian low income cut-offs (26).

**Table 7:** Catheter provision by jurisdiction compared to recommendations of the Canadian Urological Association indicates widespread inadequacies in public support for catheters for those with SCI in each province in Canada.

Province/Program	Policy documentation regarding public funding for catheters for individuals living with SCI	CUA recommends *single use* and *hydrophilic coated catheters* for those with neurogenic bladder function	
		Single-use supported by policy? (quantity allowed by policy)	Hydrophilic catheters supported by policy?
BC	Quantity limits based on medical prescription: “The least expensive, appropriate medical or surgical supplies may be provided to eligible clients in order to avoid an imminent and substantial danger to health.”	N	N
AB	“Intermittent catheters and condom catheters - combined total cannot exceed 70 every 2 months … Clients are responsible for all costs resulting from an upgraded product choice”.	N (35/month)	N
SK	Full coverage of “Incontinence and medical supplies (including catheter supplies”	N (4/day)	Unclear; possibly with prescription
MB	“Home Care services may include … medical supplies and equipment”	N	N
ON	Attendant care service provider “is responsible for providing the supplies the person requires”	N (~1/day)	N
QC	“Material aids are provided by this program based on the specific needs of the person”	N	N
NB	“Only the most cost-effective means of meeting the client's catheterization need will be approved. Brand name products will only be considered when generic products are not available or when generic products will not meet the client's medical needs. (Justification will be required) … In cases where quantities exceed monthly maximums, justification from a health professional will be required to consider exceptions.”	N (4/day)	N
NS	“The most economical option, up to $200 per month, approved by Care Coordinator. Amounts exceeding $200 per month approved by Casework Supervisor”	N ($200/month)	N
PEI	“Applicants will have access to the prescription medications and medical supplies they require, whether they are related to their disability or not”.	N (# not specifically limited; limits based on sliding scale of contribution)	N
NL	“Provides basic supportive health products to individuals who meet program criteria to assist them with activities of daily living”.	N (1/day)	N
FNIHCC/NIHB	“Coverage for incontinence items, which can be either one type of product or a combination of different products, that can be dispensed every 3 months”	N (4/day)	N
VAC	“When a health professional has prescribed a benefit that appears on the benefit grids, this is generally sufficient evidence for a decision-maker to approve the benefit.”	N ($350/month)	N

As noted, the CUA recommends those with neurogenic bladder function use single-use catheters, and that hydrophilic coated catheters are recommended above single-use, non-coated catheters.

**Sources:** British Columbia Employment & Assistance (18); KI_BC_; Alberta Health (117); KI_AB_; Saskatchewan Ministry of Health (22); KI_SK_; Manitoba Department of Families (19); KI_MB_; Government of Ontario (78); KI_ON_; Gouvernement du Québec (134); KI_QC_; New Brunswick Department of Social Development (2016); KI_NB_; Nova Scotia Department of Community Services (83); KI_NS_; AccessAbility Supports (110); KI_PEI_; Newfoundland and Labrador Department of Health and Community Services (25); KI_NL_; Campeau et al. (42).

Table 7 compares each province's documented policy restrictions on quantity, type, and quality of funded catheters in relation to the best practice recommendations of the Canadian Urological Association (CUA). As indicated, quantity and quality restrictions are inconsistent with recommendations of the CUA for people living with neurogenic bladder function. For example, NB's Ostomy/Incontinence Policy states “Only the most cost-effective means of meeting the client's incontinence need will be approved. Brand name products will only be considered when generic products are not available or when generic products will not meet the client's medical needs (Justification will be required)” (27).

KIs across the country reported that when clients did not receive an adequate quantity of catheters, they either covered costs themselves, resorted to practices such as reusing catheters intended as single-use items, and/or restricted their fluid intake to reduce urine production and therefore the daily number of catheters required [KIs_BC, MB, ON, QC, NB, NL_]. KIs reported some individuals attempted to reduce their costs by purchasing and using sub-standard items (e.g., catheters with unpolished eyelets), but also returned to purchasing higher quality items after observing bleeding, or developing strictures requiring surgical repair (KI_MB_).

“*As a taxpayer, I understand why we have limitations on some of this stuff. I think it's fine to be cautious with public money, but I also think it's really important that we look at the bigger picture […] $1,000 today vs. multiple thousands down the road, I know which I would prefer, but our system doesn't see it that way.” KI*_BC_

### 3.3 Wheelchairs

#### 3.3.1 Public health care delivery for essential mobility devices for ambulation replacement ranges from loans-based equipment pools of partially complete wheelchairs to co-pay delivery models in 9/10 provinces. Public funding in Saskatchewan and federal programs approaches 100% of basic wheelchair costs, without means-testing

Documentation regarding WC supply programs was available and reviewed in each province. It indicated that health care delivery for a ‘basic' WC depends upon financial assessments and status in 4/10 provinces (BC, ON, PEI, NL; Table 8). In the remaining six provinces, loans-based programs are used, described below.

**Table 8:** Summary of essential mobility assistive devices by province: means-testing, financial implications, and limitations.

Province/Program [Means-testing? Y/N]	Overview of means testing, eligibility, and financial and functional restrictions of publicly funded wheelchair mobility equipment programs for individuals with SCI
**BC [Y]**	**Eligibility:** requires financial assessment (means-tested), pre-approval, and no other family resources. Must be receiving EAPD and spend down assets for eligibility**. Implications:** single individuals with no dependents receive 100% funding for WC and mobility equipment with annual income ≤$16,200. No coverage for income >$16,200. Equipment restricted to least expensive deemed to meet mobility need.
**AB [N]**	**Eligibility:** medical justification (not means-tested). **Implications:** WCs are loaned from an equipment pool (WC remains property of AB Health) or ordered through approved vendors if suitable WC unavailable. WC pool limited to < 5 models. Clients can choose a grant in lieu (≤$3,900) for an “*Upgrade*” status WC. Client covers remaining costs and retains ownership of WC and components.
**SK [N]**	**Eligibility:** medical justification (not means-tested). **Implications:** WCs are loaned through SAIL by the Ministry of Health. Limited to < 5 models. Clients may opt for trial “*grant-in-lieu*” program to receive a $2,500 grant for the purchase of WC. Client covers remaining costs and owns the WC and its components.
**MB [N]**	**Eligibility:** medical justification (not means-tested). **Implications:** WCs are loaned through non-governmental non-profit charity organization with funding provided by provincial government. Limited to < 5 models. WCs remain property of charity organization (Manitoba Possible). Clients cover costs of any allowed upgrades and return WCs in unmodified state.
**ON [Y]**	**Eligibility:** ON residents who have a disability requiring a mobility aid for 6 months or longer are eligible for 75% coverage of eligible and pre-approved essential assistive devices under ADP. Clients receiving specific financial supports receive 100% coverage through ADP. **Implications:** WCs must be on preapproved list, which includes multiple manufacturers and models. Cost-sharing model with greater relative choice.
**QC [N]**	**Eligibility:** prescription (not means-tested). **Implications:** WCs are loaned (remain property of provincial government). WC models must be on preapproved list (not publicly available). Equipment ordered through provincial rehabilitation centers.
**NB [N]**	**Eligibility:** medical justification (not means-tested). **Implications:** loans program operated through Easter Seals with funding from provincial government. If no suitable WC in existing equipment pool, WC can be ordered. Model restrictions not specified. WC must be returned if new WC is requested.
**NS [N if** ** < 65 y Y if** **≥65 y]**	**Eligibility:** prescription and determination of financial need through assessment of income, assets, and associated costs. Clients 65 and older must undergo means testing for DSP eligibility. **Implications:** ADP equipment loans program operated through Easter Seals with funding from provincial government. Available equipment list not publicly viewable.
**PEI [Y]**	**Eligibility:** annual income < $119,708. **Implications:** medical equipment is covered through AAS, with copayments required based on a sliding scale of annual income. If AAS funds WC to ≥ 75% coverage, it remains property of AAS and must be returned if no longer needed.
**NL [Y]**	**Eligibility:** based on calculation of “Needs Test” (means testing) involving consideration of income, RRSP/RRIF contributions, and allowable expenses. **Implications:** WCs become property of the individual. Models are restricted by supplier availability (not preapproved list Needs Test calculations are complex, requiring significant time and professional involvement, following a flowchart with multiple decision points.
**NIHB [N]**	Eligible NIHB clients receive 100% coverage with no means-testing when equipment is not otherwise covered. This can lead to delays when disagreements arise over which public entity will provide coverage. Eligibility requires client be a First Nations person registered under the *Indian Act*, an Inuk recognized by an Inuit land claim organization, or a child < 2 years old whose parent is an NIHB client. Equipment must be prescribed by a health professional and provided by a supplier.
**VAC [N]**	Veterans eligible to receive treatment benefits under the *Veterans Health Care Regulations* are eligible to receive equipment. This includes, amongst many other eligibilities, Veterans with a disability directly related to their service. Eligible clients are generally approved for coverage of items outlined in benefits grid. Items not listed in benefits grid may be eligible for coverage if prescribed, supported by research as effective, and validated by a health care professional.

For ease of comparisons, main assumptions, calculations, and maximums that would be paid for attendant services are based on calculations for a single person living alone with SCI.

SCI, spinal cord injury; EAPD, employment and assistance for persons with disabilities; SAIL, Saskatchewan aids to independent Living; EIA, employment and income assistance; ADP, assistive devices program; ODSP, Ontario disability support program; DSP, disability supports program; AAS, AccessAbility supports program; RRSP, registered retirement savings plan; RRIF, registered retirement income fund; SAP, special assistance program; NIHB, non-insured health benefits for First nations and inuit; VAC, veterans affairs Canada; KI, key informant.

(a) While provincial health policies outline details for coverage and eligibility, coverage generally requires medical justification and/or prescription by professionals and is restricted to the most inexpensive options available which will meet basic need. (b) KI_*NL*_ reported that in practice, those calculating expenses for those needing WCs are relatively understanding of the significant costs associated with SCI, such that most clients receive 100% coverage for their WC unless their income is relatively high or they have private coverage.

**Sources:** British Columbia Employment & Assistance (128); British Columbia Ministry of Health (116); KI_BC_; Alberta Health (21, 32, 36, 117, 129–132); KI_AB_; Saskatchewan Ministry of Health (22, 34); KI_SK_; KI_MB_; Manitoba Possible (33); Manitoba Department of Families (19); Government of Ontario (20, 28, 78, 119); KI_ON_; KI_QC_; Gouvernement du Québec (80, 133, 134); Societe de l'assurance automobile du Québec (135); New Brunswick Department of Social Development (35, 86, 136); Ability New Brunswick (38); KI_NB_; Nova Scotia Department of Community Services (83, 137); KI_NS_; Easter Seals Nova Scotia (138); AccessAbility Supports (23, 31, 107, 108); KI_PEI_; Newfoundland and Labrador Department of Health and Community Services (25, 122); KI_NL_; Indigenous Services Canada (139–142); VAC (143–149, 151); Government of Canada (11).

Cost-sharing requirements vary in the 4/10 provinces that means test, as do restrictions on eligible WC models and components, quantity and quality of equipment, and replacement eligibility schedules (Tables 8, 9). Sliding scales for co-pay fees and financial eligibility restrictions are used in PEI and NL (23, 25). In ON, each person requiring a WC for at least 6 months is eligible to receive up to 75% of costs for WCs and components on a selected and curated list, with upper limits on funding levels provided [e.g., $3,098 for a rigid manual wheelchair (MWC)]. If the person is receiving social assistance in ON they receive 100% coverage of WCs (28). Eligibility for publicly funded WC equipment in BC requires recipients be receiving social assistance and requires a prescription and financial assessment (29). Of the 4/10 provinces that means-test, an eligible product and model list exists only for ON (30). The other provinces (BC, PEI, and NL) do not provide a list of eligible models, and do not explicitly exclude particular products or models (25, 29, 31). However, BC provides additional direction in their policy documentation stipulating that “The least expensive, appropriate medical equipment and devices may be provided to specific recipients to assist with a medically essential need. There must be no other resources available to the client to provide the medical equipment requested.” (29). Definitions for basic and least expensive appropriate WC were not available.

In the remaining six provinces, WCs are provided through WC equipment loans programs, administered either by a provincial health authority (AB, SK, QC) or by charity organizations (MB, NS, NB). In loans-based programs, product manuals containing a list of WCs eligible for temporary use are available in 4/6 provinces (AB, SK, MB and NB) (32–35). Our online review indicated that although eligible model and product lists exist for QC and NS, they are not accessible to the public. In provinces with eligible product manuals, wide discrepancies exist in the number and range of products and models funded, such that one to two models per user category are funded in MB to seven (manual) or 11 (power) eligible WC models in AB (32, 33).

Means-tested and loans-based WC programs allowed partial grants for self-funded upgrades “*in lieu*” of available equipment in AB, SK, and ON (34, 36, 37). In other provinces, equipment upgrades and modifications are discouraged, and persons receiving WCs must sign a waiver stating that if they modify the WC to meet their unique functional and ergonomic needs, they will return the WC to the loaned equipment pool in its original form (KI_MB_). Replacement schedules, order and repair wait times, and eligible sub-components varied widely by jurisdiction (Table 9). Except for NB, we were unable to identify documented or publicly shared information on typical wait times for WCs, from initiating contact with provincial WC programs to time of delivery and initial set-up of WC equipment. Documentation from NB indicated that wait times varied by funder (faster for insurance-based funders, such as worker's compensation schemes, and slowest for those needing to rely on public support) and ranged from 2 months to more than 3 years (38). For all provinces, WC quality is intended only to meet basic functionality within the home, and does not consider education, workplace, exercise, or weather and terrains for outdoor functionality.

“*They're not doing a fulsome assessment around what a person needs, they're doing an assessment of what the government would agree to, right? And it's flawed in its limitation to therapy. It does not focus on functional recovery, it only focuses on the ability to manage in your home.” KI*_ON_

**Table 9:** Summary of essential mobility devices funded in each provincial or federal jurisdiction.

Province/Program	Wheelchair program types, equipment and component eligibility, replacement schedules, wait times, and repair eligibility
BC	Wheelchairs—Purchased and administered by provincial government - WC model, restrictions, and eligible components: restricted to least expensive equipment prescribed and deemed medically essential to achieve basic mobility or positioning; list of models/manufacturers not publicly available; backrests eligible as a positioning device. - Wait times ~ 6–12 months for assessment by occupational therapist, plus >2 months for manufacture and delivery. - Repairs: lengthy delays reported for repairs; restricted to equipment/components funded by province; self-funded upgrades ineligible. - *Replacement:* WC replacement timeline based on medical need; cushions eligible every 2 years - Recreational, lift, and backup chairs ineligible. Bathroom, transfer, positioning, and floor/ceiling lift devices may be eligible with medical justification.
AB	Wheelchairs—Loans program, administered by provincial government - WC model, restrictions, and eligible components: models restricted (< 5) by a few manufacturers; list publicly available; MWC maximum $3,900 (AB portion); cushions and backrests eligible. - Clients required to attend seating clinic for assessment - Wait times ~2 months for assessment, plus ~3–4 months for WC manufacturing and delivery - Repairs: restricted to equipment/components funded by province; self-funded upgrades ineligible - *Replacement:* PWCs minimum 7 years, MWCs every 5 years, cushions and backrests minimum 3 years - Clients responsible for rental costs while waiting for assessment and delivery of equipment. Bathroom, lifting, and transfer devices may be covered with medical justification.
SK	Wheelchairs—Loans program, administered by provincial government - WC model, restrictions, and eligible components: restricted (< 5 models) by a few manufacturers; list publicly available; cushions eligible as a loaned component; backrests and adaptive seating options may be loaned if requisitioned by licensed specialist - Eligibility: requires referral by health care professional, funder of last resort (must be ineligible from other agencies/sources) - Clients cannot opt to pay for upgrade features or components - Repairs: lengthy delays reported; equipment is generally aging and often refurbished - *Replacement:* WCs replaced if clients have significant change in need or repair is uneconomical. Bathroom, transfer, and lifting devices loaned to clients. Grants are available for home and vehicle modifications.
MB	Wheelchairs—Loans program, administered by charity organization Manitoba Possible - WC model, restrictions, and eligible components: restricted (< 5 models) from 1–2 manufacturers; list publicly available; funding maximums not indicated; cushions eligible through SCI MB - Physical assessment of need and application form must be completed by an OT, specialization in WC mobility not required - Backrests ineligible unless client is receiving EIA - Wait times: lengthy delays in referrals (≥6–12 weeks), assessment of eligibility and approval of request (≥6–12 weeks), plus ≥12 weeks for manufacture and delivery if not available in equipment loans pool, and initial set up (~2–6 weeks). Total wait times typically ~ 6 months, although waits of >40 weeks have been reported; wait times reported as a significant factor in rehabilitation hospital discharge delays for recent SCI. - Repairs: restricted to equipment/components funded by province; self-funded upgrades ineligible; lengthy delays reported - *Replacement*: WCs replaced based on need; expected to last 5 years; basic cushions eligible ( ≤ $600, every 3 years). Loans program—WC must be returned if new equipment is requested. Client may keep chair as “back up” if it is damaged and considered “irreparable”. Bathroom, transfer, and lifting devices considered for coverage if client is receiving home care or EIA.
ON	- WC model, restrictions, and eligible components: equipment must be on pre-approved list of eligible models and components (e.g., backrests, cushions); upgrades or different features not on approved list are ineligible; maximum approved funding level listed for each item [e.g. MWC < $3,900 (ON portion)]; multiple manufacturers and multiple distinct WCs approved compared to eligible model limitations in other provinces (e.g., MB, SK, and AB). - WCs must be ordered through approved list of vendors - *Replacement:* PWCs, MWCs, and components eligible for replacement if needs have changed and device no longer appropriate or if unable to be repaired economically; cushions eligible every 2 years. Transfer/lift devices ineligible.
QC	Wheelchairs—Loans program, administered by provincial government - WC model, restrictions, and eligible components: models must be on pre-approved list; list not publicly available; eligible models reported as generally most inexpensive available, although clients may apply for equipment outside provincial list with medical justification; cushions and postural supports eligible. - Upgrade features and components funded with medical justification - A backup WC is eligible if client is working or attending school - *Replacement*: eligible for new chair when repair cost becomes >80% of the cost of a new WC - Equipment must be returned if new equipment is requested. Bathroom, transfer, and lifting devices are eligible for coverage. Funding for home modifications available; wait list for program up to 2 years. Funding for vehicle modifications available.
NB	Wheelchairs—Loans program, administered by charity organization Easter Seals - WC model, restrictions, and eligible components: if WC not available in equipment pool, it may be ordered by Easter Seals; model restrictions are not specified, list of available models not publicly available. Rather, limits are $8,500–$12,000 for power, $3,000 for folding frame MWCs and $6,000–$7,500 for rigid frame MWCs; cushions and seating/positioning aids are eligible for funding - Clients must be assessed and have equipment medically justified - *Replacement*: WCs minimum 5 years; cushions and back supports minimum 3 years; - Wait times: lengthy wait times reported (from 2 months to >3 years); referral and prescription reported taking up to 2 years. Bathroom, transfer, and lifting devices eligible for funding support.
NS	Wheelchairs—Loans program, administered by charity Easter Seals - WC model, restrictions, and eligible components: clients apply to receive new or refurbished chairs (as well as mobility aids and assistive devices) from an equipment pool; list not publicly available; must be “*most economical option available*” - Requires prescription by occupational therapist - *Replacement*: information not publicly available. Medical equipment recommended by a health care practitioner may be approved under DSP. Bathroom and bedroom safety equipment may be available through a donated equipment pool.
PEI	- WC model, restrictions, and eligible components: WC eligibility based on medical justification by health care practitioner; if ≥75% of WC is funded by AAS, it remains the property of AAS and must be returned; funding maximums not indicated; cushions and positioning equipment are eligible. - Dependent upon assessment at the provincial seating clinic - *Replacement*: information not publicly available - Wait times reported as significant. Bathroom, transfer, and lifting devices are eligible for coverage. Funding for home and vehicle modifications available.
NL	- WC model, restrictions, and eligible components: models are restricted to supplier availability, not restricted to an approved list; WCs are prescribed and features/components are eligible based on medical justification; WCs become the property of the individual; funding maximums not indicated; cushions and backrests covered with justification - Clients must attend seating clinic for assessment - *Replacement*: information not publicly available - Wait times: WCs ~3–4 months. Bathroom, lifting, and transfer devices are eligible for funding support.
**NIHB**	- WC model, restrictions, and eligible components: WCs are prescribed and all features/components are eligible based on medical justification; WCs must be most cost-effective option to meet medical needs; eligible equipment list has not been updated in a number of years; modern safety features and components may not be covered - Backup MWCs provided to PWC users - *Replacement*: eligible for replacement every 5 years (manual and power), unless item is no longer working or meeting client's needs due to change in medical condition; cushions and backrests eligible every 3 years - Repairs: providers are expected to maintain and repair equipment under warranty - Wait times: Errors made in the application process can lead to delays or denial of funding. Bathroom, transfer, and lifting devices are eligible for funding support.
**VAC**	- WC model, restrictions, and eligible components: WCs are prescribed, and all features/components are eligible based on medical justification - WCs become the property of the client - Funding limited to type and model which most reasonably addresses mobility needs - *Replacement*: MWC ≤ $4,000 eligible every 3 years; PWC ≤ $9,000 eligible every 3 years; Sport/exercise WC ≤ $4,000 eligible every 10 years; Shower WC ≤ $2,000 eligible every 2 years; Transport WC ≤ $350 eligible every 5 years; Cushions ≤ $800 eligible every 2 years; Custom seating/backrest devices ≤ $3,000 eligible every year - Items may be purchased, rented, or leased (with an option to purchase) - Repairs: VAC covers repairs upon request - Backup MWC may be eligible for power chair users. Bathroom, transfer, and lifting devices eligible for funding support, including installation fees. Funding for home and vehicle modifications may be available.

SCI, spinal cord injury; EAPD, employment and assistance for persons with disabilities; SAIL, Saskatchewan aids to independent living; EIA, employment and income assistance; ADP, assistive devices program; ODSP, Ontario disability support program; DSP, disability supports program; AAS, AccessAbility supports program; RRSP, registered retirement savings plan; RRIF, registered retirement income fund; SAP, special assistance program; NIHB, non-insured health benefits for First Nations and Inuit; VAC, veterans affairs Canada; KI, key informant; PWC, power wheelchair.

(a) Provincial/Delegated health policies outline details for public support and eligibility, support generally requires medical justification and/or prescription by professionals, typically restricted to the most inexpensive option that meets basic mobility needs. (b) KI_NL_ reported that in practice, staff calculating expenses for those needing WCs are relatively understanding of the significant costs associated with SCI, such that most receive 100% funding for their WC unless their income is relatively high, or coverage is available through private insurance.

**Sources:** British Columbia Employment & Assistance (29, 128); British Columbia Ministry of Health (116); KI_BC_; Alberta Health (21, 32, 36, 129–132, 150); KI_AB_; Saskatchewan Ministry of Health (22, 34); KI_SK_; KI_MB_; Manitoba Possible (33); Manitoba Department of Families (19); Government of Ontario (20, 28, 30, 85); KI_ON_; KI_QC_; Gouvernement du Québec (80, 133, 150); Societe de l'assurance automobile du Québec (135); New Brunswick Department of Social Development (35, 136); Ability New Brunswick (38); KI_NB_; Nova Scotia Department of Community Services (83, 137); KI_NS_; Easter Seals Nova Scotia (138); AccessAbility Supports (23, 31, 107, 108); KI_PEI_; Newfoundland and Labrador Department of Health and Community Services (25, 122); KI_NL_; ISC (139–142); Veterans Affairs Canada (143–149); Government of Canada (11).

Significant concerns regarding mobility equipment were reported by KIs, including ill-suited and inadequate equipment without consideration of lifestyle or functional improvements that may occur in the initial years postinjury. Further concerns included a lack of any coverage after age 65, excessive wait times, and problematic and non-transparent provision via charity organizations, sometimes with aging, refurbished equipment. KIs also reported that self-funded upgrades to WC sub-components or self-purchase of other commercially available models are often necessary to reduce risk of injury and enable safe community participation outside the home [KI_MB_]. In provinces where eligibility for public funding of specific mobility devices is tied to social assistance, KIs stated that clients must undergo repeated financial assessments and may be required to spend down assets to become eligible for public support [KIs_BC, MB_].

“*If you're not on any kind of social assistance […] finding the 25% yourself… I think a lot of people fall through the cracks. And I mean, we know how expensive it is when you have a spinal cord injury and all the equipment that you have to have. It's so difficult to get coverage and it's just not equitable across the board. So there are a lot of people who are managing with subpar equipment because they just can't afford to pay the co-payments for the type of equipment that they need.” (KI*_ON_*)*

### 3.4 Common themes from KI interviews: substantial financial and medical hardship in obtaining SCI-related health care in Canada

KI responses were collated, summarized, and organized by six themes partitioned into their respective domains (Themes 1 through 4), with Theme 5 relating mainly to inadequate accessible housing and Theme 6 reported limitations and inadequacies that could broadly be categorized as fitting within a “Social Determinants of Public Health” framework. As can be seen (Table 10), KIs reported widespread inadequacies in public funding for SCI-specific services, supplies, and mobility equipment. There are significant difficulties in recruiting and retaining trained attendant care workers, multiple disincentives to re-entering the workforce following SCI, and multiple physical and health-care related barriers to returning to a person's home community. Overall, KIs reported that for those living with the chronic medical condition of SCI, policies relating to provision of essential health care across each of the three domains led to considerable financial hardship and emotional distress in every province within Canada.

“*The biggest barrier to any person […] with a disability, it's not so much the disabling condition. People adapt and learn and move their lives on. But it's the poverty that people experience now that can be soul-destroying.” KI*_PEI_

**Table 10:** Summary of main themes reported by key informants.

Theme 1—Attendant service limitations (A) Significant difficulty in recruitment/retention of attendant care workers “The safety issues in New Brunswick arise due to the home support worker shortage. We have a lot of people that have workers that aren't showing up.” KI_NB_									
Provinces with similar concerns									
BC	AB	SK	MB	ON	QC	NB	NS	PEI	NL
x				x		x	x		
(B) Attendant services limited to self-managed care in rural settings “I'd say anybody living in a more rural area has a lot more difficult time accessing any kind of Home Care Services. And like I mentioned before, the vast majority that I know still that live rurally access self-managed care.” KI_AB_									
Provinces with similar concerns									
BC	AB	SK	MB	ON	QC	NB	NS	PEI	NL
x	x				x		x		
Theme 2—Medical supply limitations (A) Short sighted policies that either do not provide or provide inadequate numbers of catheters to meet CUA's best practice recommendations . “There just isn't the coverage for people to access the supplies that they need and so they end up using generic supplies. They don't have enough of them, so they're reusing them, and it's probably causing more UTIs and other issues that could easily be prevented. And you know, let's face it, the cost to the health care system is probably a lot higher because there isn't that coverage.” KI_ON_									
Provinces with similar concerns									
BC	AB	SK	MB	ON	QC	NB	NS	PEI	NL
x	x	x	x	x	x	x	x	x	x
(B) Limitations lead to non-sterile reuse of catheters intended for single-use and/or restriction of fluid intake “These things should be covered for everybody below a fairly high income level because it costs so much out of pocket, and we know people are making decisions that maybe aren't the best in terms of reusing or limiting liquid to make their catheter box last longer.” KI_BC_									
Provinces with similar concerns									
BC	AB	SK	MB	ON	QC	NB	NS	PEI	NL
x			x	x	x	x			x
Theme 3—Medical supply and wheelchair/equipment limitations Short-sighted policies either do not provide or provide inadequate or inappropriate medical supplies and wheelchair mobility equipment, leading to increased long-term health care costs and decreased quality of life “[Limitations in supply provision] prevents people from accessing gainful employment, it prevents people from accessing social participation, and it creates social isolation, and creates increase of infection. […] this can be a spiraling affect [to] the overall health and well being of an individual.” KI_ON_ “As a taxpayer, I understand why we have limitations on some of this stuff. I think it's fine to be cautious with public money, but I also think it's really important that we look at the bigger picture […] $1,000 today vs. multiple thousands down the road, I know which I would prefer, but our system doesn't see it that way.” KI_BC_									
Provinces with similar concerns									
BC	AB	SK	MB	ON	QC	NB	NS	PEI	NL
x	x	x	x	x					
Theme 4—Wheelchair equipment limitations “Medical justification” based on assessment and/or prescription is required for wheelchair coverage, yet may not involve the expertise and functional needs of the user “[Limitations with provision of essential supplies] restricts individuals to actually choose, or make their right to self-determination on what they require, especially when it limits choice.” KI_AB_									
Provinces with similar concerns									
BC	AB	SK	MB	ON	QC	NB	NS	PEI	NL
x	x	x	x	x	x	x	x	x	x
Theme 5—Limitations arising from inadequate accessible housing, attendant service, and/or mobility and transfer equipment (A) Combination of inadequate housing, lack of transfer devices, and/or inadequate attendant service hours leads to unhygienic and/or undignified experiences with primary outcome of client restriction to bed baths “Those most in need are the ones who are not getting their needs met.” KI_NS_									
Provinces with similar concerns									
BC	AB	SK	MB	ON	QC	NB	NS	PEI	NL
x	x	x	x	x	x		x		
(B) Reliance on long term care resulting from shortage of accessible housing and/or care services “Health authorities are not very creative in helping people find ways to stay in the community. It seems like the default is facility care even when that's not what the person wants or what would be best for them.” KI_BC_									
Provinces with similar concerns									
BC	AB	SK	MB	ON	QC	NB	NS	PEI	NL
x		x	x						x
(C) Lack of accessible housing exacerbates discharge wait times, limits access to attendant services, and cuts people off from social support of their home community “We have a lot of people who cannot go back to their home community because the housing isn't accessible.” KI_MB_									
Provinces with similar concerns									
BC	AB	SK	MB	ON	QC	NB	NS	PEI	NL
x			x					x	x
Theme 6—Limitations fitting within a ‘Social Determinants of Public Health Framework' (A) Disincentive to leave social assistance due to loss of coverage for essential health care. “Any individual that has an injury that don't have another source of income that depends on our government system got no other choice but to live in poverty.” KI_NL_ “The biggest barrier to any person […] anybody with a disability, it's not so much the disabling condition. People adapt and learn and move their lives on. But it's the poverty that people experience now that can be soul-destroying for people.” KI_PEI_ “We know how expensive it is when you have a spinal cord injury and all the equipment that you have to have. It's so difficult to get coverage and it's just not equitable across the board. So there are a lot of people who are managing with subpar equipment because they just can't afford to pay the co-payments for the type equipment that they need.” KI_ON_									
Provinces with similar concerns									
BC	AB	SK	MB	ON	QC	NB	NS	PEI	NL
x	x	x	x	x	x	x	x	x	x
(B) Inequality inherent in each domain of the health care system, and clients who have the knowledge, time, energy, confidence, capacity, transportation, and funding required to advocate for themselves are more likely to self-advocate change and receive services they require “It does take some advocacy […] You have to be ready to fight for what you want, or they'll roll all over you.” KI_QC_									
Provinces with similar concerns									
BC	AB	SK	MB	ON	QC	NB	NS	PEI	NL
			x	x	x				
(C) Health care system is failing to meet the needs of people living with SCI “The home support system was set up for the needs of seniors, and that is the majority of where the home support hours go because we have lots of seniors. But they're not necessarily meeting the needs of other people with more significant disabilities.” KI_BC_ “We don't support anyone very well with significant disabilities and that's one of the reasons why there's still so many problems with people with disabilities being integrated into society, because if the basic needs aren't being met effectively by our systems, whichever one it might be, then people are going to have more difficulty being involved in recreation or family events or work or any of those other things.” KI_BC_ “I would say that there is gross inadequacies in the public-based system compared to these insurance-based systems because there's such a severe curtailing of what they will provide and what hoops you have to go through in order to be able to have the devices that are there […] they're just not assessed from an actual true functional perspective.” KI_MB_ “They‘re not doing a fulsome assessment around what a person needs, they're doing an assessment of what the government would agree to, right? And it's flawed in its limitation to therapy. It does not focus on functional recovery, it only focuses on the ability to manage in your home.” KI_ON_ “We have a lot of people in New Brunswick choosing medical assistance in dying because they cannot get the supports they need from the disability support program. Access to specialists such as pain management specialists is also failing.” KI_NB_									
Provinces with similar concerns									
BC	AB	SK	MB	ON	QC	NB	NS	PEI	NL
x	x	x	x	x	x	x	x	x	x

KI, key informant; UTI, urinary tract infection; SCI, spinal cord injury.

Key Informants; British Columbia Employment & Assistance (18, 128); Alberta Health (117); Saskatchewan Ministry of Health (76); Manitoba Department of Families (19); Manitoba Possible (33); Government of Ontario (78); Nova Scotia Department of Community Services (83); Newfoundland and Labrador Department of Health and Community Services (25).

## 4 Discussion

This is the first comprehensive evaluation of public funding for medically necessary SCI-related services, supplies, and equipment in Canada. We identified a heavy reliance on means-testing to limit public funding within each domain of essential need, as well as widespread inconsistencies and discrepancies in the degree to which each province implemented means-testing and calculated funding support. We also identified inadequacies for SCI-related services, supplies, and equipment.

### 4.1 Attendant services

Currently, policy documentation indicated that 5/10 provinces and both federally managed health attendant services programs (AB, MB, ON, QC, PEI, ISC, and VAC) do not means-test to determine eligibility for attendant services. The remaining five provinces which perform means-testing use widely varying funding calculation strategies for determining user co-pay fees (BC, SK, NB, NS, and NL). In 2/5 provinces which implement copayments for attendant services, fees are based on both hours of need and income (SK and NS), whereas fees in the remainder of provinces (BC, NB, and NL) are based on income calculations alone. Where maximum annual fees exist (and none is stated for NB), they range from $3,600 to $27,000, representing between 7% and 23% of calculated income (see Table 4). In our modeling, it also became apparent that co-pay fees are triggered at very low calculated annual income levels, ranging from $10,632 in NB to $41,511 in BC for a single person living alone with SCI.

Although we did not perform a sensitivity analysis for these calculations to enable them to be more widely generalizable to families with multiple members and/or incomes, they do accurately reflect the wide range of fees charged for attendant services in the five provinces that means-test in Canada. Finally, our assessment of attendant services was solely for ADL and was restricted to toileting, bathing, dressing, and getting in and out of bed. It excluded assessment of other services such as laundry and cleaning, since our initial assessments indicated these services were rarely, if ever, publicly funded under any provincial schema.

KIs reported the use of means-testing with cut-offs for support at extremely low levels of income leads to a considerable financial disincentive to seek employment, particularly for entry level jobs. These financial considerations may reflect additional strains within Canada relating to social determinants of health for those living with SCI in provinces that limit funding for attendant services based on income levels. KI-reported system-wide deficiencies include a lack of properly trained care workers, inadequate levels of service - particularly within rural or Indigenous settings - and a lack of transparent quality assessment tools to evaluate whether levels or quality of services met the functional needs of those living with SCI in any province. Our goal was mainly to identify discrepancies in policy and funding for attendant services between provinces in Canada, and not to specifically assess objective or validated measures of adequacy of attendant services, but we did note that most provinces reported using various assessment tools for prescribing levels and hours of service such as the internationally recognized interRAI (Resident Assessment Instrument) framework (39). In future, it may be of interest to assess the extent to which each province follows validated frameworks and assesses quality of service provision within the attendant service domain for ADL.

Although we were unable to find policy reviews specifically relating to SCI-related long term attendant services for ADL, we did identify comparative policy and assessment reports for long term in-home attendant services more generally, such as for older adults in Sweden and Finland, summarized below.

As outlined in the 2021 WHO World Knowledge Center Policy Series on Long Term Care No. 8: Sweden (40), Sweden has a national legal framework for long term care that stipulates publicly financed and high-quality services be available to all citizens according to need rather than ability to pay, and therefore no means-testing criteria are applied to the provision of care. Provision of services is organized and provided at the municipal level. In 2008, Sweden implemented an Act on Free Choice Systems that allowed municipalities to implement provider choice, wherein users have choice between authorized private and public providers for home care services. Municipalities have the legal obligation, autonomy, and can levy and collect taxes to provide services that fulfill long term care needs, with a best practice focus on remaining in the home.

Since 2007, the Swedish Government established and has used an Open Comparisons national quality monitoring system for long term care and grades care providers' performance based on 28 indicators of quality of care. Relative comparisons between municipalities are based on a traffic light system. Differences at the municipal level do exist, but not in whether ability to pay limits access to publicly funded services. Rather, municipal variation relates to opting to implement the Free Choice system, or in the degree to which each municipality allows benefits to be paid to family members to provide care within the home. KIs in our study reported that certain provinces allowed or disallowed family members to provide services. Thus, it appears that Sweden has implemented federal policies regarding provision of in-home long-term care, that it be provided without consideration for financial need, and that it monitors quality of service provision. Overall, this seems quite different from the organization of attendant services in Canada.

Although not directly comparable, a recent review of user/co-pay fees for home care services for ADL and medical help for older adults in Finland indicated that despite national legislation indicating that care services should be available to all based on individual needs regardless of social and financial status or place of residence, a single person living alone would be required to pay, on average, 16%, 20%, or 30% of their gross income in user fees to receive 4, 8, or 28 hours per month of home care, respectively (41). To be comparable for a person with SCI, only the case involving 28 service hours per month (~1 hour per day) would be relevant, since a person with SCI would typically require daily services for ADL (typically 10–15 hours per week or 40–60 per month). Furthermore, the lower hours per month (4 and 8) include services for laundry, house cleaning and help with other tasks, which are typically ineligible in Canada. Nonetheless, wide variation was seen in the range of user fees by municipality from 2.5% to 24% for 4 hours; to 3%−30% for 8 hours; to 15%−35% for 28 hours per month of care, assuming a gross monthly income of 1,500 Euros (41). It is unknown if different fee structures are used for those with SCI in Finland, and so the results are not necessarily comparable.

### 4.2 Neurogenic bladder and bowel supplies

Policy documentation indicates means-testing is used to limit access to neurogenic bladder and bowel supplies in 9/10 provinces. Outside of AB and SK, funding for neurogenic bladder and bowel supplies is restricted to those Canadians with very limited income. As summarized in Table 7, current levels of public funding and documented quality restrictions for SCI-related medical supplies contravene expert and evidence-based opinion. The CUA recommends single-use and hydrophilic catheters for those with neurogenic bladder syndrome to decrease infection and tissue damage rates, which requires at least 6–8 hydrophilic catheters per day, assuming a conservative estimate of bladder emptying once every 3–4 hours (42). None of the provinces meet this recommendation, with catheter limits ranging from 1 per week (NL) to 4 per day (SK & NB) ((22, 27), KI_NL_).

Policy documentation in three provinces included language specifically restricting supplies to the “most economical option available” (BC, NB, and NS; Table 7). In most provinces where policy did not comment on economy of supplies, KIs reported that the quality of funded catheters was a significant concern (KI_AB, MB, ON, QC, NL_). Their concerns are supported by the research literature, which indicated that sub-standard catheters without sufficient lubrication and polished eyelets can tear the epithelium in the urethra, causing bleeding and tissue damage which can lead to scarring (43). Tears in the epithelium increases risk of urinary tract infection and scarring and can lead to the need for surgery to unblock urethras narrowed by scar tissue (43, 44). Infections and surgeries increase the burden on the health care system, and as such, opting for supplies which are “the least expensive and appropriate for the purpose” (18) may lead to a greater overall health care cost. Cost analysis in Italy supports the cost-effectiveness of single-use hydrophilic-coated catheters for those with neurogenic bladder dysfunction, especially once downstream health care costs of not using single-use hydrophilic catheters are considered (45).

“*As a taxpayer, I understand why we have limitations on some of this stuff. I think it's fine to be cautious with public money, but I also think it's really important that we look at the bigger picture […] $1,000 today vs. multiple thousands down the road, I know which I would prefer, but our system doesn't see it that way.” KI*_BC_

As an essential daily medical supply, catheters can represent a significant expense to individuals over their lifespan. In addition to contravening best practice recommendations, KIs report that reuse of single-use catheters is often considered unhygienic, inappropriate, and increases risk of infection, while the restriction of fluid intake to “make do” with limited numbers of catheter supplies can adversely affect health.

Finally, 7/10 provinces refer to catheters and associated supplies as “*incontinence*” products, with only three referring to them as “*medical*” supplies (BC, QC, and PEI). Such wording not only diminishes the medical necessity of these items, KIs report it is also considered humiliating and moves the medical need for the device into the social realm (KI_MB_), thereby placing the onus of securing these items on the individual rather than within the health care domain. We are currently investigating public policy in comparable countries and our preliminary evidence from KIs in Australia, Denmark, Germany, the Netherlands, Slovenia, and Sweden indicate that single-use and hydrophilic coated catheters are considered standard of care and funded through their respective health authorities (46).

### 4.3 Essential mobility devices

Our systematic review of policy documentation indicated that despite each province having access to the same models of commercially available WCs and sub-components, we observed wide discrepancies in funded equipment, with very little systematic evidence-based planning and oversight, program delivery assessment, or quality control mechanisms. The requirement in some provinces for individuals to be receiving social assistance to remain eligible for coverage of essential mobility devices (e.g., BC) appears at odds with the Canadian government's adoption of universal health care policy, as does a financial requirement to spend down assets to become eligible for social assistance (and therefore, coverage of mobility devices).

In certain provinces, the contrast between public coverage of mobility and prosthetic equipment is significant. For example, BC explicitly excludes WCs from the Fair PharmaCare policy, in which personal expenses to purchase high cost items are capped at a yearly maximum based on a sliding scale of net family income (e.g. annual family net income of $30,000 requires a family deductible of $650 with a family maximum of $900) (47). This program funds prosthetics and orthotics, breast, eye, or ear replacements, smoking cessation products, and even drugs for medical assistance in dying (MAiD) (48, 49). Another contrast regards ownership of the medical device such that items including coronary stents, glucose infusion pumps, prosthetics, orthotics, and hip or knee replacements are fitted to and become the property of the individual. Unlike these devices, WCs are not individualized and are not the property of the user and provided as a loaned item in 6/10 Canadian provinces.

Wide discrepancies were also observed in a recent review of Canadian public health policy for prosthetics, including variability in eligibility criteria, funding levels, and replacement and repair intervals, which reached similar conclusions that “many persons with limb loss are forced to rely upon personal resources, fundraising, or the charity of non-governmental organizations in order to meet this basic health care need” (50). Howard et al., also acknowledged wide variation in the number and types of devices deemed to achieve “basic functionality” and concluded “there is no indication that that criteria are appropriately inclusive or restrictive, or if they were developed with the input of stakeholders” (50). Some provinces supported the need for and funded ‘advanced' devices (e.g., microprocessor knees) but with widely varying levels of support ($5,000 to $17,690 in 2019 pricing). Other provinces did not provide funding for advanced components but did allow residents to select an advanced device in lieu of a basic device (MB and ON) (50). Finally Howard et al., stated stakeholders reported confusion in navigating governmental systems, similar to our findings regarding navigating attendant services for ADL, attaining neurogenic bladder and bowel management supplies, and WC mobility devices.

We compared Canadian policy to international best practice guidelines, whitepapers, and fitting and testing standards for MWCs. Overall, there is an understanding that optimizing choice in model selection and unique fitting/modification is most likely to meet the user's unique functional needs and this process should be central when identifying adequate WC programs (51). “It is imperative that clinicians and equipment service providers understand the importance of matching the equipment to the user and invite active participation from the user to identify specific needs. Evaluating and recommending MWCs should focus on the functional potential of the user and their long-term goals, rather than reimbursement circumstances alone” (52). Given that WC customization improves efficiency, increases comfort, and reduces negative outcomes (53) while promoting community involvement, independence, social inclusion, and overall well being (54, 55), WCs must be fitted and customized on an individual basis (53). Thus, WC programs in Canada that offer minimal choice in models and which do not include backrest or seating components will not meet these minimal international functional requirements (e.g. BC and MB), whereas those offering more choice and include necessary sub-components are more likely to do so (e.g. AB, SK, and ON), although research is needed to objectively evaluate this.

White paper and best practice guidelines recognize that WC weight should be minimized. As reducing the mass of MWCs has been shown to lower push rim forces which may reduce injury risk (56), ultra-lightweight WCs (< 30 pounds) should be provided to optimize propulsion and limit repetitive strain injury (52). Given that pressure injuries and orthopedic deformities can result from poorly configured WCs and their postural components, it is critical that WCs include adequate seating (such as air cell-based cushions) and solid backrests (as opposed to the minimally supportive upholstery backs) (54). Furthermore, “A properly configured WC ensures that risks of secondary impairments from poor positioning are minimized and promote the overall participation and quality of life for the active SCI user thereby reducing medical care costs” (54). In Canada, each province includes individual measurement and fitting, but several deny or limit customization and/or upgrades of subcomponents which would provide positioning and ergonomics to reduce injury, prevent orthopedic deformities, and enable better mobility within the community.

Finally, timely repairs are critical for maintaining function. Given that adverse consequences of WC breakdown include being stranded, injured, missing work, school, or appointments, and increased risks of pressure injuries and rehospitalization, WC quality should be considered a priority (57–60). Furthermore, preventative maintenance should be performed routinely, and as adverse consequences are exacerbated by lengthened wait times without back up equipment, WC repair must occur in a timely manner while WC users must have access to back up equipment. Our study indicated that timely repairs and adequate replacement equipment were an unmet need and a critical concern reported by KIs in many provinces (Table 9).

Different countries use a variety of means to address their universal WC equipment delivery policies. A review of regulation and funding modalities for mobility aids (WCs, walkers, and crutches) compared program mandates and eligibility criteria, and included devices and coverage in Germany, Italy, Netherlands, Norway, and the UK (61). All countries stipulated universal coverage for mobility aids, although variation existed between countries in balancing federal versus regional organization of delivery systems and mechanisms (61). For example, Germany, the Netherlands, and Norway do not involve charities in any aspect of WC supply, whereas Italy and some countries within the UK do. In Germany, benefit packages are issued by directives from federal joint committees of key stakeholders (physicians, hospitals, insurers, etc.), which identifies which patients are entitled to WC assistive technology benefits and establishes the fundamentals of a benefit catalog for assistive devices, including listings of individual products across 33 categories that can be provided at government expense (62). Similarly, other countries such as Italy set out National Ministerial Decrees “that explain in detail who is entitled to an assistive device and under which conditions”. Germany also limits out of pocket expenses for assistive technology to 2% of annual income, or 1% for qualified patients with chronic medical conditions (61). Countries such as Ireland, the United Kingdom, Netherlands, and Switzerland consider a WC an essential need for those unable to ambulate, and provide these mobility devices universally (51, 63, 64). Appropriate selection of equipment requires input of experts in fitting, design, and use of these specialized mobility devices (51, 65–69). Additionally, many countries fund basic car and home adaptations as they are considered an essential need and delivered universally (63, 64).

### 4.4 Reported effects on those living with SCI in Canada – themes revealed by KI Interviews

As noted in Table 10, there were several recurrent themes in many provinces relating to perceived inadequacies in health care delivery in each domain. In some cases, KIs indicated that lack of access to necessary resources was so drastic as to trigger certain individuals to access MAiD.

“*We have a lot of people in New Brunswick choosing medical assistance in dying because they cannot get the supports they need from the disability support program.” KI*_NB_

Health care delivery in all domains is generally considered inadequate, and many Canadians with SCI choose to self-fund attendant care, supplies, and equipment, or attempt to live with sub-standard health care. While some provinces fund greater quantities or higher quality medical supplies and equipment, KIs in several provinces report systems focusing on cost rather than function, and report individuals are required to advocate for what they would consider minimal levels of services, supplies, or equipment, forcefully articulate their functional needs, and take responsibility to obtain necessary additional prescriptions.

Such reports appear to differ from policy and delivery of other forms of health care in Canada, in which items such as cardiovascular stent implantation or hip or knee replacements are provided without undue hardship or advocacy on the individual's behalf to obtain a minimally functional device. Given that the United Nations has determined that access to WC equipment and supplies for bladder emptying are fundamental human rights (70, 71), it appears that Canada may not meet these basic UN requirements. It is unclear why Canada fails to meet these international obligations as it relates to these SCI-specific health care needs.

“*I would say that there [are] gross inadequacies in the public-based system compared to these insurance-based systems because there's such a severe curtailing of what they will provide and what hoops you have to go through in order to be able to have the devices that are there […] they're just not assessed from an actual true perspective.” KI*_MB_

### 4.5 Study limitations and future research

Our policy review initially reflected online data from 283 websites between September 2021 through January 2022. We re-accessed and updated as many links as possible, with four remaining broken (March 2026). Our observations related to means-testing, attendant services, and quantity and quality of supplies and equipment remain valid, and formulae used to calculate financial need and public funding have not changed substantively through January 2026, nor have conclusions or funding levels within each domain in the intervening time period.

Our observations regarding eligibility, assessment, and access to attendant services, neurogenic bladder and bowel supplies, and WC and related equipment were all based on publicly available documentation which was present for each domain in each province (Table 1). Thus, we have confidence in our assessments regarding eligibility, means testing calculations, and quantities and qualities of these services supplies and equipment in each province. Outside of the requirement to be under 65 for program eligibility in many provinces, there were only three domains/provinces in which we identified a complete absence of public funding programs for those living outside of the social welfare system: neurogenic bladder supplies in ON, MB, and BC and WC equipment in BC. We are confident that our observations of the absence of public programs accurately reflect the situation in these provinces because we triangulated our data with multiple KIs and additionally communicated with SCI physiatrists and rehabilitation professionals. Further, the fact that we were successful in identifying motor vehicle and workers compensation insurance-based coverage programs in these provinces for these items suggests our methods were adequate.

In the domain of WC equipment, we are confident in our observations and assessments regarding the numbers and types of models of WCs and sub-components funded in each province in which policy manuals exist (AB, SK, MB, ON, and NB). We were unable to estimate the number and range of products and sub-components funded in provinces lacking publicly available product manuals (BC, QC, NS, PEI, and NL). Nonetheless, our overall findings and conclusions that there is a lack of transparency and an absence of documented policies, procedures, and evidence-based decision-making when considering WC equipment to replace lost mobility remain valid.

The financial modeling we included is recognized to have limitations in that the models were based on assumptions of a single person with SCI living alone with no dependants and all income assumed to be “earned income”; that is, with no portion coming from areas such as the guaranteed income supplement, survivor's allowance, income assistance programs, disability assistance programs, or a war veteran's allowance. The calculations were based on a hypothetical individual and therefore cannot be generalized to other income situations or to family settings with multiple people living in one dwelling with or without additional earners contributing to calculated family income. The complex and varied formulae between provinces and domains led to challenges in sensitivity analyses which were decided to be beyond the scope of this paper. However, while not broadly generalizable, we believe that the financial outcomes of a hypothetical individual are useful in making between-province comparisons and in illustrating the relatively high costs for health services to perform ADL after SCI. Finally, we did not model the combined costs in each province to the individual when considering all three domains and this would be of interest in future for better understanding total relative costs to individuals living with SCI in different provinces.

As we were unsuccessful in recruiting KIs within the Yukon, Northwest, or Nunavut Territories, we were unable to evaluate SCI-related health care policies in these locations and our analysis was limited to the provinces. Although our sample of KIs is relatively small at 16, each KI had been working in their field for at least 2 years and had received multiple hours of training in these specific public policy domains. Thus, it is unlikely our results are influenced by KIs being unaware of eligibility and service delivery in their geographical regions(s). Further, in addition to working in SCI rehabilitation, 10/16 KIs had lived experience with SCI and would be keenly aware of relevant policies as they would be users of this health care.

As the study covered a broad range of health care domains with complicated delivery mechanisms, KIs did not always have expertise in all areas. We addressed this by interviewing additional KIs in regions where KI expertise was lacking. Some questions were intentionally subjective and open-ended to optimize the likelihood of identifying program limitations. Due to resource limitations, we relied on a small sample size which may not reflect the full range of experiences in each province. Additionally, actual practice data gathered from KI interviews sometimes challenged or contradicted policy data. We addressed this by repeated cross-referencing with policy documents. Finally, statements about the effect(s) of public policy on people with SCI are quoted directly and were not independently verified.

Within each domain of need, variety exists in the administrative gate-keeping and in relative access to services, supplies, and equipment, based on financial need. Overall, obtaining medical services and supplies essential for life after SCI appears bureaucratic and subject to repeat and invasive financial assessments and oversight on the part of medical or administrative professionals. In some domains and provinces, such professionals may not have appropriate SCI-related experience and/or training needed to properly match functional needs with equipment. For example, in ON, the prescribing OT or PT for a WC must have a specialization in wheeled mobility, whereas in MB such specialization is not required. These variations appear more striking when compared to accessing other medical devices or treatments considered essential in Canada, in part because they are not subject to any financial assessments, such as kidney dialysis, insulin pumps, or cardiac stents. It is unclear what policy-based rationale is behind these differences within the context of a universal Canadian health care system. This is a question for further study, as it may be relevant within the contexts of health equity, human rights, and social determinants of health. Future research involving policy makers and health economists could systematically and quantitatively examine whether funded items and costs are appropriate, and if administrative costs are either appropriate or disproportionate to the cost of items provided. Research could include a cost-benefit analysis of either provision vs. non-provision of health care within these three domains that considers practices in European and other G7 and similar countries that ascribe to universal and publicly funded health care systems.

## 5 Conclusions

These findings indicate there are widespread discrepancies and inadequacies in SCI-related health care delivery between provinces in Canada. There is also a lack of clarity in each province's calculus for determining levels of public funding within each of the three domains examined. The reliance on means-testing appears to contradict Canadian universal health care policy and represents inequity within a framework of social determinants of health. When compared to other countries with universal health care frameworks, it appears that Canada may not provide similar levels of public support for attendant services, neurogenic bladder supplies, and WCs. These findings suggest the need for the development of a national SCI-related health policy framework guided by standards which are based on objective expert opinion from key stakeholders and current research. Ideally, such standards would be informed by non-partisan scientific review processes and involve key stakeholders, including policy makers, public administrators, health care financial analysts, SCI rehabilitation professionals, and lived experience experts in wheeled mobility.

## Data Availability

The raw data supporting the conclusions of this article will be made available by the authors, without undue reservation.
