# Supplementary material for: Review and assessment of policies and practices for spinal cord injury-related health care supplies, services, and mobility equipment reveal wide inequities and inadequacies in Canada
Source: Front Public Health. 2026 Apr 13;14:1762879. doi: 10.3389/fpubh.2026.1762879 (PMC13111559; doi:10.3389/fpubh.2026.1762879)
Supplement: Supplementary file 1 [file Data_Sheet_1.pdf]

APPENDIX 1 KEY INFORMANT INTERVIEW GUIDE

Research will focus on the following three essential spinal cord injury-specific needs 1) Personal attendant care for ADL; 2) Essential neurogenic bowel and bladder management supplies; and 3) Essential mobility assistive devices (power and manual wheelchairs for those unable to ambulate).

Specific Items within each area of need:

1) Personal attendant care for ADL will focus on the following: neurogenic bowel management routine; neurogenic bladder management routine; dressing (clothing); and bathing.
2) Essential neurogenic bowel and bladder management supplies will be: catheters [intermittent & indwelling (Foley or condom drainage), stoma/urinary diversion supplies]; ostomy supplies; nonsterile examination gloves; lubricant; and suppositories.
3) Essential assistive devices will be: power wheelchairs; manual wheelchairs; lifting/transfer devices (e.g., Hoyer lifts); and essential seating for pressure relief for power and manual wheelchairs.

As experts in these areas you will likely have documentation regarding the delivery of these services and supplies within your jurisdiction. As part of our environmental scan, we would very much appreciate any and all documentation available. So, as you go through the questions and you are reminded of documentation/URLs etc. that you use, please provide this information.
A) Please describe your employment role(s) relating to assisting persons with SCI in obtaining the above-noted essential services and supplies:
B) Please indicate if you are a person with lived SCI-experience in obtaining the above-noted essential services and supplies: Yes or No
C) Either per year, per month or per week, on average, how often do you help individuals with SCI obtain these supplies/services (approximately).
D) Recognizing that these questions cover broad areas of service or equipment delivery, as you go through these questions, please let us know your level of expertise and/or indicate if you don't feel comfortable in commenting due to a lack of familiarity.

Questions regarding each item within each area of need:

1) Public provision of personal attendant care for ADL
With respect to personal attendant care for ADL:

- How are each person's care needs determined and prescribed and who is responsible for the assessment?
- Does your jurisdiction have a 'delegated care act'? If so, please describe.
- Are there options for self/family-managed care delivery of these services? If so, how is this process organized and delivered? What are the eligibility criteria? How portable is self/family-managed care (i.e., what happens when a person moves)?

- What are the different service delivery models available for home care (supported housing, congregate care such as 'Fokus' units in MB or the 'Hub and spoke' model in ON)? If so, please list and describe, including the process for determining eligibility and limits/restrictions in terms of levels/extents of service?
- What is the process to determine hours of care needed? Is there a formal or standardized assessment (e.g., InterRAI)? If so, please describe as appropriate for: initial discharge from in-patient SCI rehabilitation stay; when needs change in the community.
- Is there an upper limit of hours at which point the person would require institutional or other forms of supported care (e.g., grouped care settings)? If the limits vary, please indicate.
- Is there a cost-sharing income-based component to delivery of these services? If so, please describe.
- How are these services provided in your jurisdiction, who is permitted to deliver each service and are there restrictions on service delivery (e.g., only a nurse can change an indwelling catheter; a home care aid can provide bathing services but a home support worker cannot)?
- Is there an appeal process if the person disagrees with either the type or level of service? If so, please describe.

With respect to neurogenic bowel management routine; neurogenic bladder management routine; dressing (clothing); and bathing:

- What specific services are eligible?
- Are there time limits per service?
- Are there restrictions in the level of service?
- What is the process if there is a nonscheduled emergency event that requires care?
- Is the level and extent of service generally considered adequate?

Can you comment on any differences in level of service or limits to services depending upon rural versus urban or other living situation?
Does the publicly funded system state that it adheres to the Independent Living Philosophy Act? In practice, does the publicly funded system adhere to the Independent Living Philosophy Act (i.e. does not limit # of showers per week).

2) Publicly funded essential neurogenic bowel and bladder management supplies

With respect to essential neurogenic bowel and bladder management supplies:

- What are the specific supplies that are eligible?
- Are there limits to the number provided during a given time period? If so, express as unit per day/week/month/year.
- Are there restrictions based on supplier or individual's circumstance (e.g., income based, limits or restrictions on the type of product)?
- Are there certain essential medical supplies that are publicly funded but only in certain circumstances (e.g., catheters if a person is receiving homecare)?
- In your opinion, what are the limitations/concerns with provision of these essential GU supplies?

3) Public coverage for essential mobility assistive devices

With respect to essential assistive devices:

- What are the specific components/features eligible (e.g., tilt or height raising feature on power chairs)?
- Which components/features are only eligible for public coverage based on a specific additional justification (e.g., tilt feature on power chairs)?
- What are the specific model restrictions on publicly funded manual or power chairs (e.g., only Quickie Xperience2 power wheelchairs is funded)?
- Are additional features available as 'add-ons that are self-funded or co-paid' for either power or manual wheelchairs (e.g., raise feature on power chair)? If so, please list. Describe the process and funding model for ordering the original and upgraded components.
- What essential mobility/care devices are not funded at all (e.g., Hoyer lift systems, transfer boards, bath seats)?
- Are there certain essential equipment items that are publicly funded but only in certain circumstances (e.g., wheelchair cushions, indwelling catheters if a person is receiving home care)?
- Regarding your assistive device program, can you provide us with a 100% 'funded' list of devices.
- Is there a list of items that are subsidized on a 'copay' basis? If so, please provide.
- Where does the public go to learn this information?
- In your opinion, what are the limitations/concerns with provision of these essential assistive mobility devices?

4) Nonpublic Coverage

What are the inequities between the public and insurance-based systems? This includes provincial insurance-based (motor vehicle and worker's compensation based) or federally-funded (NIHB and Veterans Affairs) systems. If you can, provide limits, inclusions/exclusions, additional coverages, eligibility criteria, etc., as they relate to personal attendant care for ADL, essential neurogenic bowel and bladder management supplies, and essential assistive devices.

<u>Motor vehicle insurance-based systems (e.g., MPI).</u> Indicate if these are torte-based or no-fault. If a mixed system, please state the circumstances that determine whether under torte-law vs. needs-based provision.

<u>NIHB-based coverage</u>
Do you provide counseling services to Indigenous peoples – If so – we would like to learn about how the provision of these essential services differs for this population.

Any other comments regarding how you would compare these nonpublic insurance-based providers to each other?

Any other comments regarding how you would compare these nonpublic insurance-based providers to the public payor?

Finally, how would you rank them? Are some/one better than others in terms of their coverage?
