# Supplementary material for: Review and assessment of policies and practices for spinal cord injury-related health care supplies, services, and mobility equipment reveal wide inequities and inadequacies in Canada
Source: Front Public Health. 2026 Apr 13;14:1762879. doi: 10.3389/fpubh.2026.1762879 (PMC13111559; doi:10.3389/fpubh.2026.1762879)
Supplement: Supplementary file 2 [file Data_Sheet_2.pdf]

Table A2
Public Availability of Program Information for Attendant Services for ADL

Province/Program	Program Information Accessible to the Public[a]
**British Columbia**	https://www2.gov.bc.ca/gov/content/health/accessing-health-care/home-community-care/care-options-and-cost/home-support<br>Eligibility, services, process to access, cost (not easily navigated, although regulations are referenced)
**Alberta**	https://www.albertahealthservices.ca/assets/info/seniors/if-sen-home-care-brochure.pdf<br>Eligibility, services, assessment, contact information
**Saskatchewan**	https://publications.saskatchewan.ca/api/v1/products/22841/formats/29232/download<br>Outlines eligibility, services, cost, assessment, and all policies, although lengthy and relatively difficult to navigate
**Manitoba**	https://www.gov.mb.ca/health/homecare/guide.pdf<br>Eligibility, assessment, services, contact information, appeals. However, specific details regarding the assessment process and tools and the levels of service provided are not documented
**Ontario**	https://www.ontario.ca/page/home-community-care<br>Eligibility, services, process to access, contact information, appeals/complaint process
**Quebec**	https://www.ciusss-ouestmtl.gouv.qc.ca/en/care-and-services/seniors-residents-and/or-those-experiencing-a-loss-of-independence/home-support-services<br>Basic eligibility, services
**New Brunswick**	https://socialsupportsnb.ca/en/program/disability-support-program<br>Eligibility, services, process to access, contact information
**Nova Scotia**	https://novascotia.ca/dhw/ccs/home-care.asp<br>Basic eligibility, services, contact information, appeals/complaints process
**Prince Edward Island**	https://www.princeedwardisland.ca/sites/default/files/publications/provincial_home_care_program_brochure.pdf<br>Eligibility, services, process to access, contact information
**Newfoundland & Labrador**	https://www.gov.nl.ca/hcs/files/personsdisabilities-pdf-home-support-program-client-handbook.pdf<br>Eligibility, services, support team members and plan development, contact information, appeals process
**FNIHCC**	https://www.sac-isc.gc.ca/eng/1582550638699/1582550666787<br>Eligibility, services, contact information.
**VAC**	https://www.veterans.gc.ca/eng/housing-and-home-life/help-at-home/veterans-independence-program<br>Basic eligibility, services, application options, additional information, related programs, frequently asked questions.

Abbreviations: ADL, activities of daily living; FNIHCC, First Nations and Inuit home and community care; VAC, Veterans Affairs Canada.

a) These references provide starting points and contact information for clients attempting to navigate a complex system. The information is sometimes vague, difficult to find, or unavailable.
INITIAL SOURCES: British Columbia Home and Community Care (2020); British Columbia Ministry of Health (2022); Alberta Health Services (2017); Government of Saskatchewan (2024); Saskatchewan Ministry of Health (2023); Manitoba Health (2019); Government of Ontario (2022); Gouvernement du Quebec (2022); Social Supports New Brunswick (2023); Nova Scotia Department of Health and Wellness (2018, 2021); Health PEI (2024); Newfoundland and Labrador Department of Health and Community Services (2005, 2024); Indigenous Services Canada (2023b); Veterans Affairs Canada (2024). Links validated and updated January 12. 2026, and were all functioning: therefore these dates may not reflect current online documentation versions and publication dates.
